# Warm versus cold blood cardioplegia in paediatric congenital heart surgery: a randomized trial

**DOI:** 10.1093/ejcts/ezad041

**Published:** 2023-02-17

**Authors:** Serban Stoica, Helena J M Smartt, Rachael Heys, Karen Sheehan, Terrie Walker-Smith, Andrew Parry, Richard Beringer, Iakovos Ttofi, Rebecca Evans, Lucy Dabner, Mohamed T Ghorbel, William Lansdowne, Barnaby C Reeves, Gianni D Angelini, Chris A Rogers, Massimo Caputo

**Affiliations:** Bristol Royal Hospital for Children, University Hospitals Bristol and Weston NHS Foundation Trust, Bristol, UK; Bristol Trials Centre, Bristol Medical School, University of Bristol, Bristol, UK; National Institute for Health Research Bristol Biomedical Research Centre, University Hospitals Bristol and Weston NHS Foundation Trust and University of Bristol, Bristol, UK; Bristol Trials Centre, Bristol Medical School, University of Bristol, Bristol, UK; National Institute for Health Research Bristol Biomedical Research Centre, University Hospitals Bristol and Weston NHS Foundation Trust and University of Bristol, Bristol, UK; Bristol Royal Hospital for Children, University Hospitals Bristol and Weston NHS Foundation Trust, Bristol, UK; National Institute for Health Research Bristol Biomedical Research Centre, University Hospitals Bristol and Weston NHS Foundation Trust and University of Bristol, Bristol, UK; Bristol Trials Centre, Bristol Medical School, University of Bristol, Bristol, UK; National Institute for Health Research Bristol Biomedical Research Centre, University Hospitals Bristol and Weston NHS Foundation Trust and University of Bristol, Bristol, UK; Bristol Royal Hospital for Children, University Hospitals Bristol and Weston NHS Foundation Trust, Bristol, UK; Bristol Royal Hospital for Children, University Hospitals Bristol and Weston NHS Foundation Trust, Bristol, UK; Bristol Royal Hospital for Children, University Hospitals Bristol and Weston NHS Foundation Trust, Bristol, UK; Bristol Trials Centre, Bristol Medical School, University of Bristol, Bristol, UK; National Institute for Health Research Bristol Biomedical Research Centre, University Hospitals Bristol and Weston NHS Foundation Trust and University of Bristol, Bristol, UK; Bristol Trials Centre, Bristol Medical School, University of Bristol, Bristol, UK; National Institute for Health Research Bristol Biomedical Research Centre, University Hospitals Bristol and Weston NHS Foundation Trust and University of Bristol, Bristol, UK; Bristol Heart Institute, University of Bristol, Bristol, UK; Bristol Royal Hospital for Children, University Hospitals Bristol and Weston NHS Foundation Trust, Bristol, UK; Bristol Trials Centre, Bristol Medical School, University of Bristol, Bristol, UK; National Institute for Health Research Bristol Biomedical Research Centre, University Hospitals Bristol and Weston NHS Foundation Trust and University of Bristol, Bristol, UK; National Institute for Health Research Bristol Biomedical Research Centre, University Hospitals Bristol and Weston NHS Foundation Trust and University of Bristol, Bristol, UK; Bristol Heart Institute, University of Bristol, Bristol, UK; Bristol Trials Centre, Bristol Medical School, University of Bristol, Bristol, UK; National Institute for Health Research Bristol Biomedical Research Centre, University Hospitals Bristol and Weston NHS Foundation Trust and University of Bristol, Bristol, UK; Bristol Royal Hospital for Children, University Hospitals Bristol and Weston NHS Foundation Trust, Bristol, UK; Bristol Heart Institute, University of Bristol, Bristol, UK

**Keywords:** Paediatrics, Cardiac surgery, Cardiopulmonary bypass, Cardioplegia temperature, Clinical trials, Randomized

## Abstract

**OBJECTIVES:**

Intermittent cold blood cardioplegia is commonly used in children, whereas intermittent warm blood cardioplegia is widely used in adults. We aimed to compare clinical and biochemical outcomes with these 2 methods.

**METHODS:**

A single-centre, randomized controlled trial was conducted to compare the effectiveness of warm (≥34**°**C) versus cold (4–6**°**C) antegrade cardioplegia in children. The primary outcome was cardiac troponin T over the 1st 48 postoperative hours. Intensive care teams were blinded to group allocation. Outcomes were compared by intention-to-treat using linear mixed-effects, logistic or Cox regression.

**RESULTS:**

97 participants with median age of 1.2 years were randomized (49 to warm, 48 to cold cardioplegia); 59 participants (61%) had a risk-adjusted congenital heart surgery score of 3 or above. There were no deaths and 92 participants were followed to 3-months. Troponin release was similar in both groups [geometric mean ratio 1.07; 95% confidence interval (CI) 0.79–1.44; *P* = 0.66], as were other cardiac function measures (echocardiography, arterial and venous blood gases, vasoactive-inotrope score, arrhythmias). Intensive care stay was on average 14.6 h longer in the warm group (hazard ratio 0.52; 95% CI 0.34–0.79; *P* = 0.003), with a trend towards longer overall hospital stays (hazard ratio 0.66; 95% CI 0.43–1.02; *P* = 0.060) compared with the cold group. This could be related to more unplanned reoperations on bypass in the warm group compared to cold group (3 vs 1).

**CONCLUSIONS:**

Warm blood cardioplegia is a safe and reproducible technique but does not provide superior myocardial protection in paediatric heart surgery.

**Figure:**
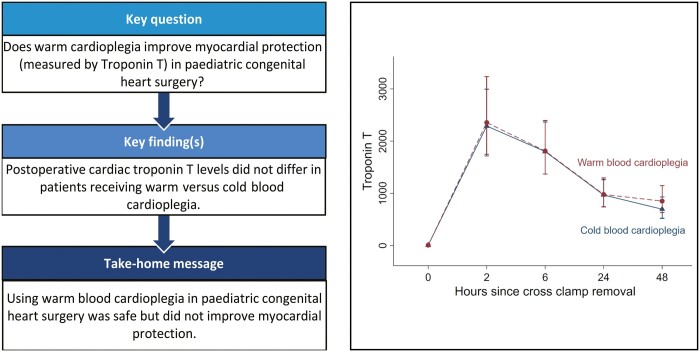

## INTRODUCTION

Myocardial protection is typically achieved via cardioplegic arrest. Several variables affect myocardial protection, including the type of crystalloid solution, mixing with blood, dose and frequency of delivery, and temperature. Sound protection and a technically adequate repair are cornerstones of surgery. Minimizing the risk of low cardiac output syndrome through adequate myocardial protection has a positive knock-on effect for other short and longer-term complications [1].

Surgical results have improved due to gains in all perioperative and intraoperative domains. However, variability in practice suggests the optimum method is unknown. Specifically regarding cardioplegia, North American centres favour the del Nido solution, whereas in the UK, St Thomas’ solution is preferred [2, 3]. The physiological evidence for cardioplegic arrest is compelling [4]. This knowledge has evolved into sound reproducible techniques, the most common being intermittent cold blood cardioplegia (ICBC). Current myocardial protection practice, however, is supported by a paucity of class I evidence. A recent systematic review identified 26 randomized controlled trials (RCTs) examining between them 12 types of cardioplegic intervention [5]. The heterogeneity of these studies prevented meta-analysis and the authors advocate further high-quality research [5].

Building on previous work, we developed an interest in techniques related to cardiopulmonary bypass (CPB) and myocardial protection [6, 7]. The previous Thermic trials showed that normothermic CPB has no deleterious effects in low-risk infants and children, a concept supported by a systematic review [8, 9]. A retrospective study showed that intermittent warm blood cardioplegia (IWBC) is safe with excellent results [6]. An RCT in 47 patients comparing cold crystalloid cardioplegia with IWBC showed that energy stores measured in myocardial biopsies have a more physiologic profile with IWBC, with no early or late neuro-developmental differences between the groups [7]. Conceptually, it makes sense to combine warm blood delivery via CPB with warm blood cardioplegic perfusion of the myocardium [1, 6, 7]. There are no studies focusing on the temperature of blood cardioplegia itself in the context of ‘warm’ CPB. We sought to address this issue [1].

## MATERIALS AND METHODS

### Ethical approval

Ethical approval was granted by the London—Central Ethics Service Committee (reference 18/LO/0205, 7 March 2018). Patients and/or parents/guardians provided written informed consent for randomization and use of their data.

### Trial design

Thermic-3 is a single-centre, parallel-group RCT. Participants were allocated in a 1:1 ratio to receive either antegrade ICBC or IWBC. Participants were followed-up for 3 months after randomization. Details of the study rationale and design are reported elsewhere [1].

### Participants and setting

Patients aged ≤18 years undergoing congenital heart surgery requiring CPB and cardioplegic arrest at the Bristol Royal Hospital for Children, a regional congenital cardiac surgery centre in the UK, were screened for eligibility. Patients weighing <3 kg, those requiring emergency surgery or secundum atrial septal defect repair as an isolated procedure were excluded. Also excluded were patients judged preoperatively by the surgeon to require deep hypothermic circulatory arrest, or deep hypothermic CPB or considered too complex [1]; patients of consenting/assenting age but lacking capacity to consent/assent and patients under the care of social services and/or where the parent/guardian was unavailable for consent.

### Interventions

Participants received either IWBC (blood cardioplegia at the same temperature as the body, ≥34°C) or ICBC (blood cardioplegia at 4–6°C) during surgery.

The route of cardioplegia infusion for both groups was usually into the aortic root, or selectively into the coronary arteries. All surgeons used the same established cardioplegia protocols based around St Thomas’ solution.

For ICBC, cardioplegic concentrate solution (Harefield Hospital Formulation, Terumo BCT Ltd) was mixed with arterialized patient blood drawn from the oxygenator, in a 1:4 ratio, to give a concentration of 24 mM K^+^ and 16 mM Mg^2+^. This was cooled for reinfusion to ∼4°C. The induction dose was 110 ml/min/m^2^, antegradely for 4 min, with a maintenance dose of 110 ml/min/m^2^ for 2 min at 20–30 min intervals.

For IWBC, the cardioplegic concentrate (Martindale sterile concentration for cardioplegia infusion) was added to arterialized patient blood via a syringe driver to achieve a concentration of 17.5 mM K+, initial infusion and reinjection rates were based on body surface area. The flow was 1–1.5 times the physiologic coronary flow (estimated at 5% of cardiac output). The arresting dose of cardioplegia was given for 1 min after electromechanical arrest of the heart. Reinjections at two-thirds of the initial injection speed were given for 1 min every 15 min during aortic cross-clamping (see Supplementary Material). CPB, anaesthetic management and all other aspects of care followed local protocols for all patients [9, 10].

### Outcomes

The primary outcome was cardiac troponin T (cTnT) over the 1st 48 postoperative hours. Secondary outcomes were cardiac function (left and right ventricular function by echocardiography), circulatory function [central venous saturations (CVS), arterial saturations, base deficit, blood lactate], blood gas and blood sample test results (pH, partial pressure of oxygen, partial pressure of carbon dioxide, c-reactive protein, haemoglobin, haematocrit, white cell count, alanine aminotransferase), renal function (serum urea and creatinine, urinary albumin and creatinine, retinol-binding protein, *N*-acetyl-β-glucosaminidase, neutrophil gelatinase-associated lipocalin), new onset arrhythmia (e.g. atrial fibrillation/flutter, ventricular tachycardia, ventricular fibrillation, nodal, junctional ectopic tachycardia or heart block), postoperative blood loss in the 1st 12 h, intubation time, time to discharge from the paediatric intensive care unit (PICU), vasoactive-inotrope score (VIS) over the 1st 48 h [11], chest and wound infections, all-cause mortality to 3 months and length of postoperative hospital stay (for further details see Supplementary Material). Reasons for hospital readmissions not listed in the protocol were coded using the Medical Dictionary for Regulatory Activities (version 24; McLean, VA).

### Sample size

The sample size was 94 patients (47/group), which provided 80% power to detect a difference of 0.46 standard deviations in cTnT, assuming correlations between pre- and post-surgery measures of 0.3 and between the post-surgery measures of 0.5, 5% statistical significance (2-tailed) and allowing for up to 15% missing data.

### Randomization

Randomization was stratified by surgical complexity [Risk Adjustment for Congenital Heart Surgery (RACHS) score <3 vs ≥3]. Random allocations were computer-generated using blocks with varying sizes, prepared by a statistician independent of the study team. Allocations were concealed until a patient was recruited and registered onto a secure purpose-designed electronic database. Randomization took place as close to the start of surgery as possible; if a participant’s surgery was unexpectedly rescheduled, they retained their randomized allocation.

### Blinding

Staff providing postoperative care were blinded to the participants’ allocation. Documents containing allocation information (e.g. anaesthetic and perfusion charts) were placed in a sealed envelope within the medical notes. Staff accessing the contents of the sealed envelope were asked to record their name and reason for access.

### Statistical methods

Analyses were based on a pre-specified statistical analysis plan and performed on an intention-to-treat basis. Continuous outcomes were compared using linear regression, time to event outcomes using Cox regression, categorical/ordinal outcomes using logistic regression and continuous longitudinal outcomes using linear mixed-effects regression. All analyses used ICBC as the reference group and were adjusted for surgical complexity (RACHS <3 vs ≥3) and baseline measures where collected. Outcomes are reported as effect sizes with 95% confidence intervals (CIs) and likelihood ratio tests were used to determine statistical significance. Further details, including pre-planned sensitivity analyses, are in the Supplementary Material. Analyses were performed in Stata version 16.1 (StataCorp LP, College Station, TX).

## RESULTS

### Recruitment

Between May 2018 and April 2020, 479 patients were screened for inclusion and 234 (49%) were eligible (Supplementary Material, Fig. S1). In total, 154 families of the eligible patients (66%) were approached and 98 (64%) consented. Of these, 97 were randomized; surgery stopped due to the Covid-19 pandemic before 1 patient could be randomized; 48 participants were allocated ICBC and 49 IWBC.

There were 29 protocol deviations (12 ICBC versus 17 IWBC, Supplementary Material, Tables S1 and S2), including 4 crossovers, 1 from ICBC to IWBC and 3 from IWBC to ICBC. The analysis population included 95 randomized participants: 2 were withdrawn before or during surgery (Supplementary Material, Table S3). Ninety-two participants were followed-up to 3 months.

### Baseline data

The median age was 1.2 years (range <1 month to 16.3 years): patients allocated IWBC were, on average, 7 months older than those allocated ICBC (median 11 and 18 months, respectively; Table 1). Overall, 34/97 (35%) participants were female. Most participants (59/97, 61%) had a RACHS score ≥3. The most common cardiac conditions were ventricular (17/97, 18%) and atrioventricular septal defects (15/97, 15%). Overall, 43/97 (44%) participants had undergone previous surgery (Supplementary Material, Table S4). Characteristics were well-balanced between groups.

**Table 1:** Demography and intraoperative details

Characteristic	Randomized to ICBC (*n* = 48), *n* (%)	Randomized to IWBC (*n* = 49), *n* (%)	Overall (*n* = 97), *n* (%)
Demography			
Age (years), median (IQR)	0.9 (0.3–5.2)	1.5 (0.3–4.0)	1.2 (0.3–4.4)
Female	12/48 (25.0%)	22/49 (44.9%)	34/97 (35.1%)
Body mass index, median (IQR)	16.1 (14.2–17.9)	15.6 (14.0–17.0)	15.8 (14.2–17.5)
Body surface area (m^2^),^a^ median (IQR)	0.4 (0.3–0.8)	0.5 (0.3–0.7)	0.5 (0.3–0.7)
Procedure			
Ross	4/48 (8.3%)	1/49 (2.0%)	5/97 (5.2%)
Pulmonary pathway procedure	6/48 (12.5%)	5/49 (10.2%)	11/97 (11.3%)
Ventricular septal defect ± atrial septal defect ± pulmonary artery debanding	9/48 (18.8%)	8/49 (16.3%)	17/97 (17.5%)
Arterial switch ± ventricular septal defect	2/48 (4.2%)	2/49 (4.1%)	4/97 (4.1%)
Tetralogy of Fallot repair	4/48 (8.3%)	8/49 (16.3%)	12/97 (12.4%)
Atrioventricular valve repair/replacement	3/48 (6.3%)	4/49 (8.2%)	7/97 (7.2%)
Atrioventricular septal defect	8/48 (16.7%)	7/49 (14.3%)	15/97 (15.5%)
Double outlet right ventricle	2/48 (4.2%)	0/49 (0.0%)	2/97 (2.1%)
Partial or total anomalous pulmonary venous drainage	0/48 (0.0%)	3/49 (6.1%)	3/97 (3.1%)
Rastelli-type procedure	1/48 (2.1%)	2/49 (4.1%)	3/97 (3.1%)
Subaortic stenosis repair	6/48 (12.5%)	4/49 (8.2%)	10/97 (10.3%)
Other^b^	3/48 (6.3%)	5/49 (10.2%)	8/97 (8.2%)
RACHS score			
1–2	18/48 (37.5%)	20/49 (40.8%)	38/97 (39.2%)
3+	30/48 (62.5%)	29/49 (59.2%)	59/97 (60.8%)
Preoperative left or right ventricular dysfunction	8/31 (25.8%)	4/31 (12.9%)	12/62 (19.4%)
Bypass data			
Duration of bypass (min),^c^ median (IQR)	101 (77.0–154.0)	96 (67.0, 136.0)	100 (69.5–146.0)
Duration of cross-clamp (min),^c^^,^^d^ median (IQR)	72 (43.0–137.0)	69 (39.0–100.0)	70 (43.0–108.0)
Mean core temperature while cross-clamp on (°C),^e^ median (IQR)	34.0 (32.3–34.8)	34.8 (32.7–35.3)	34.0 (32.5–35.0)
Cardioversion	5/48 (10.4%)	1/49 (2.0%)	6/97 (6.2%)
Defibrillation	3/48 (6.3%)	2/49 (4.1%)	5/97 (5.2%)
Ischaemic changes coming off CPB	3/43 (7.0%)	3/47 (6.4%)	6/90 (6.7%)
Lowest haematocrit during surgery (%),^a^ median (IQR)	0.28 (0.26–0.29)	0.27 (0.26–0.29)	0.27 (0.26–0.29)
Modified ultrafiltration	18/47 (38.3%)	22/49 (44.9%)	40/96 (41.7%)
Volume (ml), median (IQR)	200 (180–210)	235 (200–300)	200 (190–260)
Cardioplegia			
Intervention received^c^	46/46 (100%)	49/49 (100%)	95/95 (100%)
Temperature of first infusion (°C),^f^ median (IQR)	4.0 (4.0–4.6)	34.4 (34.0–35.0)	
Temperature of second infusion (°C),^g^ median (IQR)	4.0 (4.0–4.7)	34.2 (34.0–35.0)	
Third infusion needed	20/47 (42.6%)	35/49 (71.4%)	55/96 (57.3%)
Temperature of third infusion, if needed (°C),^h^ median (IQR)	4.0 (3.8–4.2)	34.4 (34.0–35.1)	

a Data missing for 1 patient (ICBC).

b Other procedures were: ICBC group—double outlet right ventricle and partial anomalous pulmonary venous drainage repair, aortic root replacement, tricuspid valve repair and right ventricular myectomy. IWBC group—repair of double outlet right ventricle and atrioventricular septal defect and atrial switch (hemi-Mustard), tetralogy of Fallot and atrioventricular septal defect repair, mitral valve replacement and Ross-Konno, ventricular septal defect and Ebstein’s repair, pulmonary and tricuspid valve replacement.

c Two patients (ICBC) withdrew prior to or during surgery.

d Ranges (minimum–maximum): ICBC: 25–200 min, IWBC 16–292 min.

e Data missing for 1 patient (IWBC).

f Data missing for 6 patients (4 ICBC, 2 IWBC).

g Data missing for 20 patients (13 ICBC, 7 IWBC).

h Data missing for 2 patients (1 ICBC, 1 IWBC).

CPB: cardiopulmonary bypass; ICBC: intermittent cold blood cardioplegia; IQR: interquartile range; IWBC: intermittent warm blood cardioplegia; RACHS: Risk adjustment for Congenital Heart Surgery.

### Operative details

Operative characteristics were similar in the 2 groups; the median duration of cross-clamp and CPB was 70 min and 100 min respectively, with 3- and 5-min difference between groups. The overall median duration of surgery was 225 min and blood product use was similar (Table 1 and Supplementary Material, Table S5). The median blood cardioplegia temperatures (1st infusion) were 4°C and 34.4°C in the ICBC and IWBC groups, respectively. Intraoperative cardioversion was required in 6 participants (5 ICBC, 1 IWBC) and 5 required defibrillation (3 ICBC, 2 IWBC).

### Primary outcome

**Figure 1:**
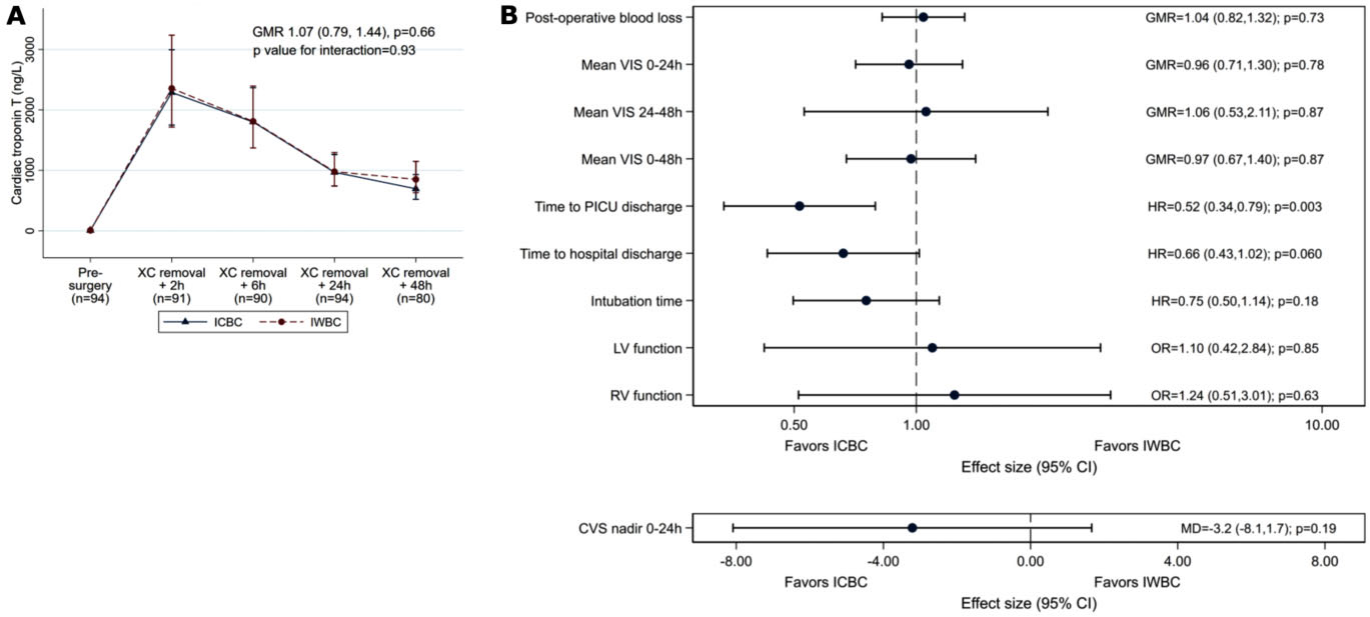
(**A**) Primary outcome: cardiac troponin T concentrations over time. Geometric mean and 95% CI of cardiac troponin T level at each time point and GMR for the effect of IWBC versus ICBC on cardiac troponin T release (95% CI). Data from 95 patients (46 allocated ICBC, 49 allocated IWBC) contributed to the analysis (missing baseline was imputed for 1 patient allocated ICBC). (**B**) Secondary outcomes. CI: confidence interval; CVS: central venous saturation; GMR: geometric mean ratio; HR: hazard ratio; ICBC: intermittent cold blood cardioplegia; IWBC: intermittent warm blood cardioplegia; LV: left ventricular; MD: mean difference; OR: odds ratio; PICU: paediatric intensive care unit; RV: right ventricular; VIS: vasoactive-inotropic score; XC: cross-clamp.

cTnT concentrations are illustrated in Fig. 1 and Supplementary Material, Table S6. cTnT concentrations increased after surgery peaking around 2 h. There was no evidence of a difference between groups [geometric mean ratio (GMR) 1.07; 95% CI 0.79–1.44; *P* = 0.66]. The pre-planned sensitivity analyses taking account of protocol deviations did not affect the conclusion (Supplementary Material, Table S6).

### Secondary outcomes

The median postoperative chest drain loss was 75 ml in both groups (GMR 1.04; 95% CI 0.82–1.32; *P* = 0.73, Supplementary Material, Table S7). Mean VIS in the 1st 48 h was also similar (median 5; GMR 0.97; 95% CI 0.67–1.40; *P* = 0.87, Fig. 1), but postoperative PICU stay was on average 14.6 h longer in the IWBC group [ICBC median 45.8 h, IWBC median 60.4 h; hazard ratio (HR) 0.52; 95% CI 0.34–0.79; *P* = 0.003]. Total postoperative hospital stay was also longer in the IWBC group (HR 0.66; 95% CI 0.43–1.02; *P* = 0.060). However, there was no evidence for a difference in duration of first intubation (HR 0.75; 95% CI 0.50–1.14; *P* = 0.18).

Electrical activity is shown in Supplementary Material, Table S8. Arrhythmias on cross-clamp removal occurred in 15/48 (31%) participants in the ICBC group and 21/49 (43%) in the IWBC group; 10 participants in each group had arrhythmias on chest closure. New onset arrhythmia postoperatively occurred in 7/47 (15%) participants in the ICBC group and 12/49 (24%) participants in the IWBC group.

**Figure 2:**
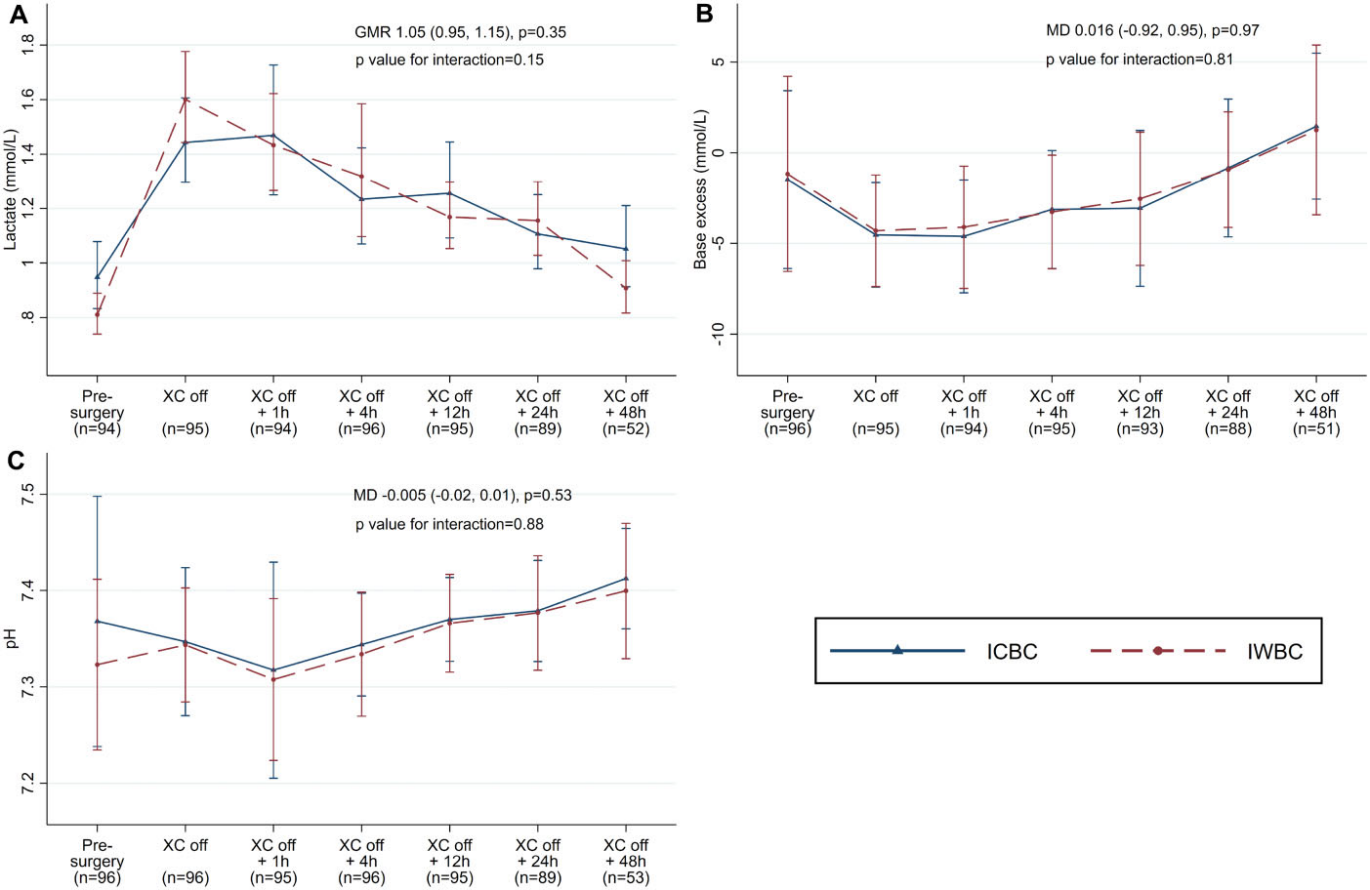
Blood gas levels over time. Geometric mean and 95% CI at each time point and GMR and 95% CI for the effect of IWBC versus ICBC on lactate (**A**). Arithmetic mean ± standard deviation at each time point and MD and 95% CI for the effect of IWBC versus ICBC on base excess (**B**) and pH (**C**). CI: confidence interval; GMR: geometric mean ratio; ICBC: intermittent cold blood cardioplegia; IWBC: intermittent warm blood cardioplegia; MD: mean difference; XC: cross-clamp.

Cardiac function data are shown in Figs 1 and 2 and Supplementary Material, Tables S9 to S11. Postoperative left and right ventricular function were similar between groups (odds ratio 1.10, 95% CI 0.42–2.84, *P* = 0.85 and odds ratio 1.24, 95% CI 0.51–3.01, *P* = 0.63, respectively). Indirect measures of cardiovascular function were also similar (nadir percentage CVS: mean difference –3.2, 95% CI –8.1 to 1.17, *P* = 0.19; lactate: GMR 1.05, 95% CI 0.95–1.15, *P* = 0.35; base excess: mean difference 0.016, 95% CI –0.92 to 0.95, *P* = 0.97), as were postoperative central arterial saturations (Fig. 1, Supplementary Material, Tables S10 and S11). Other blood gas, blood test and renal function results were also comparable (Supplementary Material, Table S11, Supplementary Material, Figs S2 and S3).

### Complications and hospital readmissions

In the period from surgery to hospital discharge, there were 185 complications reported (69 ICBC, 116 IWBC), of which 30 (5 ICBC vs 25 IWBC) were classified as serious (Table 2). There were 36 chest and wound complications in 33 participants; 21 were confirmed infections (7 ICBC, 14 IWBC, Supplementary Material, Table S12) and 10 (3 ICBC, 7 IWBC, Table 2), were considered serious. Fifteen participants underwent 19 reinterventions or other procedures prior to discharge (4 ICBC, 11 IWBC) at a median of 10 days following the study surgery. The most common reason was pacemaker insertion (Supplementary Material, Table S13). Four patients (1 ICBC, 3 IWBC) had unplanned reoperations involving CPB: revision of Rastelli pathway, atrioventricular valve repair/replacement after attempted repair and placement of a conduit after initial repair of tetralogy of Fallot with absent pulmonary valve.

**Table 2:** Complications following surgery

Complications	Randomized to ICBC (*n* = 48)		Randomized to IWBC (*n* = 49)	
	Complications, *n* (%)	Serious complications^a^, *n* (%)	Complications, *n* (%)	Serious complications^a^, *n* (%)
Total postoperative complications (events/patients)^b^	69/32 (68.1%)	5/3 (6.4%)	116/36 (73.5%)	25/12 (24.5%)
Arrhythmias				
Ventricular tachycardia/fibrillation requiring intervention	1/47 (2.1%)	0/47 (0.0%)	1/49 (2.0%)	0/49 (0.0%)
Heart block	4/47 (8.5%)^c^	0/47 (0.0%)	9/49 (18.4%)^c^	3/49 (6.1%)
Junctional ectopic tachycardia	3/47 (6.4%)	0/47 (0.0%)	5/49 (10.2%)	0/49 (0.0%)
Nodal rhythm	7/47 (14.9%)	0/47 (0.0%)	5/49 (10.2%)	0/49 (0.0%)
New pacing	8/47 (17.0%)^c^	0/47 (0.0%)	14/49 (28.6%)^c^	3/49 (6.1%)
If yes, number that became permanent	2/8 (25.0%)	0/8 (0.0%)	8/14 (57.1%)^c^	5/14 (35.7%)
Haemodynamic support^d^				
Vasodilators	8/47 (17.0%)	0/47 (0.0%)	13/49 (26.5%)^c^	1/49 (2.0%)
Pulmonary complications and treatments				
Mask continuous positive airway pressure	5/47 (10.6%)	0/47 (0.0%)	13/49 (26.5%)^c^	0/49 (0.0%)
Pneumothorax or pleural effusion requiring drainage	2/47 (4.3%)	0/47 (0.0%)	0/49 (0.0%)	0/49 (0.0%)
Chylothorax	2/47 (4.3%)	0/47 (0.0%)	0/49 (0.0%)	0/49 (0.0%)
Re-intubation and ventilation	3/47 (6.4%)	0/47 (0.0%)	7/49 (14.3%)	2/49 (4.1%)
Neurological complications				
Phrenic nerve palsy^e^	0/47 (0.0%)	0/47 (0.0%)	1/49 (2.0%)	0/49 (0.0%)
Renal complications				
Peritoneal dialysis	2/47 (4.3%)	0/47 (0.0%)	2/49 (4.1%)	0/49 (0.0%)
Gastrointestinal complications				
Necrotizing enterocolitis	0/47 (0.0%)	0/47 (0.0%)	1/49 (2.0%)	0/49 (0.0%)
Other^f^	1/47 (2.1%)	0/47 (0.0%)	0/49 (0.0%)	0/49 (0.0%)
Infective complications				
Systemic inflammatory response syndrome	4/47 (8.5%)	1/47 (2.1%)	7/49 (14.3%)^c^	1/49 (2.0%)
Respiratory infection	11/47 (23.4%)	3/47 (6.4%)	21/49 (42.9%)^g^	6/49 (12.2%)^h^
Superficial wound infection	1/47 (2.1%)	0/47 (0.0%)	2/49 (4.1%)^c^	0/49 (0.0%)
Wound dehiscence requiring rewiring or treatment	0/47 (0.0%)	0/47 (0.0%)	1/49 (2.0%)^c^	1/49 (2.0%)^c^
Other postoperative infection with antibiotic treatment	3/47 (6.4%)	0/47 (0.0%)	2/49 (4.1%)	0/49 (0.0%)
Other complications				
Pericardial effusion	1/47 (2.1%)	0/47 (0.0%)	1/49 (2.0%)	0/49 (0.0%)
Residual anatomical abnormalities (requiring surgery)	1/47 (2.1%)	1/47 (2.1%)	3/49 (6.1%)	3/49 (6.1%)

a Complications that were life-threatening or that caused hospitalization, increased length of hospital admission, persistent or significant disability or death.

b Data missing for 1 participant (ICBC).

c One participant received alternative treatment to that allocated.

d In addition, 45/47 (95.7%) participants allocated ICBC and 48/49 (98.0%) allocated IWBC received inotropes postoperatively: in 1 (IWBC), this was considered serious. Low cardiac output was recorded in the medical notes for 3/49 participants (allocated IWBC), none were considered serious: these are omitted due to the lack of a precise, pre-specified definition of low cardiac output for this study.

e No participants experienced stroke, transient ischaemic attack, paraplegia, recurrent laryngeal nerve palsy or neurological defects.

f Possibly Hirschsprung’s disease.

g Three participants received alternative treatment to that allocated.

h Two participants received alternative treatment to that allocated.

ICBC: intermittent cold blood cardioplegia; IWBC: intermittent warm blood cardioplegia.

In the period from hospital discharge to 3 months, 16 participants were re-admitted to the hospital (9 ICBC, 7 IWBC, Supplementary Material, Table S14). Eight hospitalizations were to treat a wound or chest infection (6 ICBC, 2 IWBC). No readmission was classified as likely to be related to the study intervention (Supplementary Material, Table S15). There were no deaths.

## DISCUSSION

Our results show that there is no difference in myocardial protection in the 1st 48 h when ICBC and IWBC are used, as evidenced by the similar cTnT profiles in the 2 groups. Cardiac and renal function were also comparable. However, participants in the IWBC group had on average a longer stay in PICU and experienced more complications. These events were unlikely to be linked to myocardial protection via the temperature of the cardioplegia. Specifically, surrogate markers of cardiac function, namely blood gases (including CVS), mean VIS in the 1st 48 h and predischarge echocardiography were comparable in the 2 groups. We did not include a technical performance score, which is known to directly affect outcomes [12]. The higher number of unplanned procedures in the IWBC group suggest more technical errors. Removing the 4 participants who had extended PICU stays due to requiring a reoperation reduced the median PICU stay to 45.7 h and 49.0 h in the ICBC and IWBC groups, respectively. More participants entered PICU with the chest open in the IWBC group, not a complication in itself but known to extend hospitalization. The overall rate of unplanned reoperations involving CPB is in keeping with reported rates [13, 14]. The prolonged PICU stay is likely responsible for the higher rate of infection in the IWBC group.

In Thermic-3, we aimed to extend our warm bypass investigation into myocardial protection by warm blood cardioplegia. We focused on St Thomas’ solution, which is the most commonly used preparation worldwide, and has benefitted from the highest level of scrutiny [3, 5]. The warm cardioplegia idea is not new, but has benefitted from little scrutiny. Compared to the 1 previous RCT in 47 participants, Thermic-3 is larger with more complex patients reflecting the full spectrum of care [7]. Similar to others, we have confirmed that IWBC is safe and reproducible [6, 7]. We favour ICBC for routine practice but IWBC, previously used in sporadic fashion, was easy to adopt in a trial setting. Our normal practice is to give ICBC every 20–30 min. IWBC is given in smaller doses but more frequently. This technical difference was not associated with notable differences in CPB and cross-clamp times.

### Strengths and limitations

This is one of few adequately powered RCTs focused on paediatric myocardial protection. Strengths are: inclusivity of the eligibility criteria; concealed allocation to minimize selection bias; objective outcomes and blinding to minimize detection bias, and minimal attrition. Stratification by RACHS score ensured the more complex cases were balanced across groups. A single laboratory was used for sample analysis, thereby avoiding inter-laboratory variability.

With respect to limitations, participants were recruited from a single hospital which limits the generalizability of the findings. The 2 crystalloid formulas were not identical, but they represent the most common preparations used in myocardial protection in children for cold and warm myocardial protection. They are the solutions used outside the trial setting at the study centre. There were protocol deviations, mostly related to staff being unblinded after accessing anaesthetic and perfusion records; these would be expected to have minimal impact given the objective nature of most outcomes. The 4 crossovers could have reduced the differences in outcome between groups, but there was good separation in cardioplegia temperatures reflecting adherence to the protocol. Furthermore, the crossovers (4.1%) did not reduce the statistical power; the sample size without allowance for missing data was 80 participants, allowing for 5% crossover increases the sample size to 90 participants. Overall, 95 participants contributed primary outcome data. There are limitations to the data, specifically preoperative echocardiograms rarely assessed ventricular function, thereby limiting our ability to adjust analyses for presurgery cardiac function. Fitness for discharge from PICU was not recorded reliably so actual transfer times were used, but any delays are unlikely to be differential between groups. The study has a wide range of ages and pathologies; a higher percentage of participants with RACHS score ≥3 would have further increased the applicability of the findings. cTnT is not measured routinely in clinical practice. While it provides reassurance in terms of myocardial damage, it is not a direct measurement of function. We envisaged supplementing this with a detailed echocardiographic assessment of systolic and diastolic function [1]. The pressure of clinical service delivery did not allow for research echocardiograms to be done and so we used routinely-collected echocardiogram data for contractile assessments.

## CONCLUSION

In this single-centre trial of myocardial protection with St Thomas’ solution and normothermic bypass, IWBC is a safe and reproducible technique, but it does not provide superior myocardial protection to ICBC. Whether IWBC is advantageous for the most complex patients is uncertain as they are not represented in sufficient numbers. Thermic-3 adds to the paediatric cardiac surgery evidence base and assists us in our choices of bypass and cardioplegia strategies. It remains important for surgeons to overcome difficulties related to generating high-quality randomized evidence.

## Supplementary Material

ezad041_Supplementary_Data

## Data Availability

Anonymized individual patient data will be made available upon request to the corresponding author for secondary research, conditional on assurance from the secondary researcher that the proposed use of the data is compliant with the Medical Research Council Policy on Data Sharing regarding scientific quality, ethical requirements and value for money and is compliant with the National Institute for Health and Care Research policy on data sharing. A minimum requirement with respect to scientific quality will be a publicly available pre-specified protocol describing the purpose, methods and analysis of the secondary research (e.g. a protocol for a Cochrane systematic review), approved by a UK Research Ethics Committee or other similar, approved ethics review body. Participant identifiers will not be passed on to any third party.

## Notes

Presented at the American Association of Thoracic Surgery 102nd Annual Meeting, Boston, MA, USA, 14–17 May 2022.

## ABBREVIATIONS

CIs: Confidence intervals
CPB: Cardiopulmonary bypass
cTnT: Cardiac troponin T
CVS: Central venous saturations
GMR: Geometric mean ratio
HR: Hazard ratio
ICBC: Intermittent cold blood cardioplegia
IWBC: Intermittent warm blood cardioplegia
PICU: Paediatric Intensive Care Unit
RACHS: Risk Adjustment for Congenital Heart Surgery
RCTs: Randomized controlled trials
VIS: Vasoactive-inotrope score
