# Supplementary material for: Warm versus cold blood cardioplegia in paediatric congenital heart surgery: a randomized trial
Source: Eur J Cardiothorac Surg. 2023 Feb 17;63(4):ezad041. doi: 10.1093/ejcts/ezad041 (PMC10097434; doi:10.1093/ejcts/ezad041)
Supplement: ezad041_Supplementary_Data [file ezad041_supplementary_data.zip › Supplementary Figures S1 to S3.docx]

**Supplementary Figures**

**LEGENDS:**

**Figure S 1:** Flow of participants. CPB = cardiopulmonary bypass, PIL= patient information leaflet. ^1^ Some patients may be ineligible for more than one reason, ^2^ See **Table S 3** for further details.

**Figure S 2:** Pre- and postoperative routine blood results over time. Routine blood gas (A,B) and blood test (C-G) data are presented as arithmetic means ± standard deviations (A-D) or geometric means with 95% confidence intervals (E-G). ICBC = intermittent cold blood cardioplegia, IWBC = intermittent warm blood cardioplegia, PCO_2_ = partial pressure of carbon dioxide, PO_2_=partial pressure of oxygen, XC = cross-clamp.

**Figure S 3:** Renal function: pre- and postoperative levels over time. Blood (A,B) and urinary (C-G) data are presented as geometric means with 95% confidence intervals. ICBC = intermittent cold blood cardioplegia, IWBC = intermittent warm blood cardioplegia, XC = cross-clamp.

**
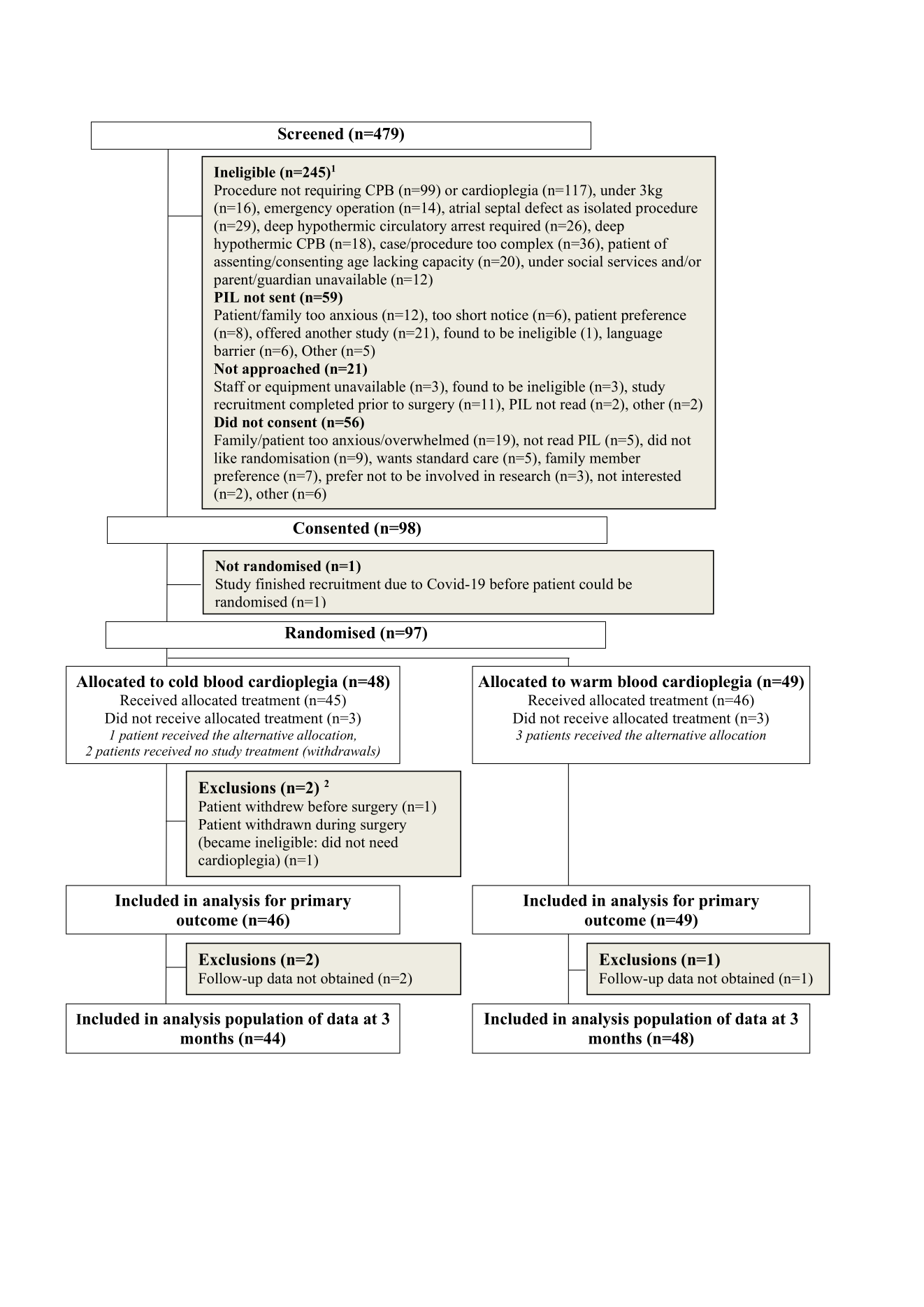
Figure S1**

**
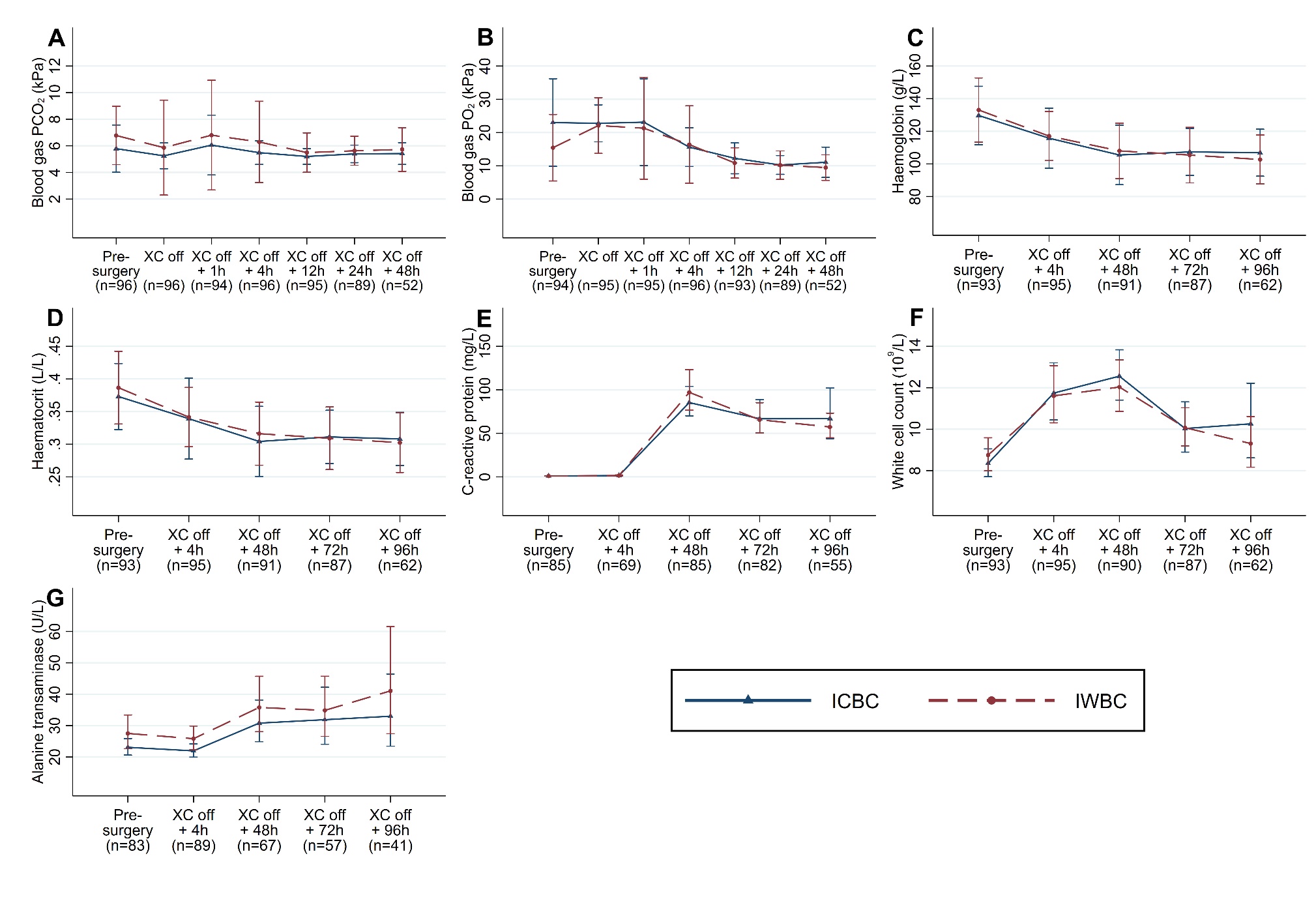
Figure S2**

**Figure S3**

**
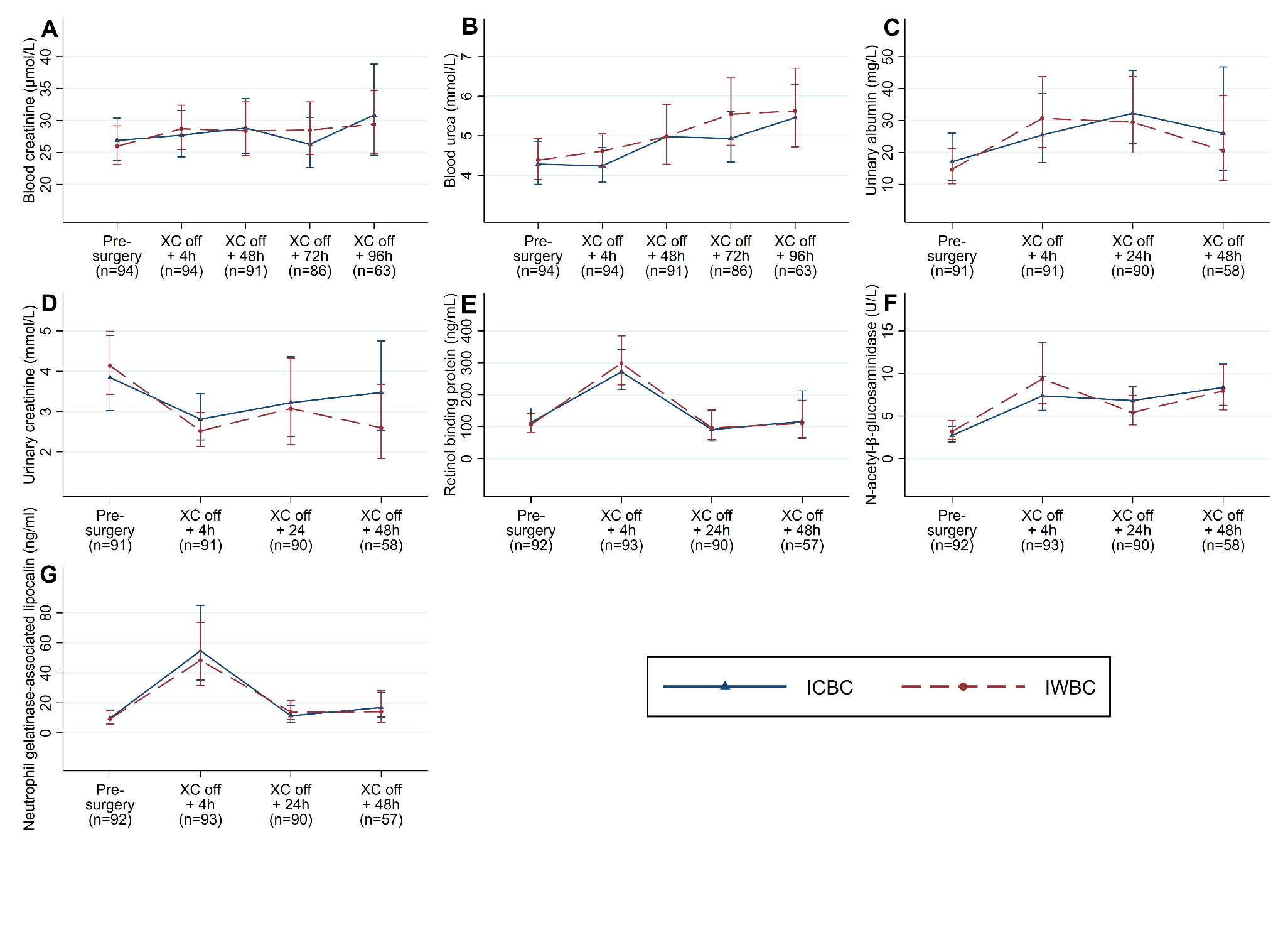
**
