# Supplementary material for: Warm versus cold blood cardioplegia in paediatric congenital heart surgery: a randomized trial
Source: Eur J Cardiothorac Surg. 2023 Feb 17;63(4):ezad041. doi: 10.1093/ejcts/ezad041 (PMC10097434; doi:10.1093/ejcts/ezad041)
Supplement: ezad041_Supplementary_Data [file ezad041_supplementary_data.zip › Supplementary Material.docx]

Supplementary Material

Contents

[1. Study interventions – further details 2](#_Toc102995102)

[2. Study outcomes – further details 2](#_Toc102995103)

[3. Statistical methods – further details 4](#_Toc102995104)

# Study INTERVENTIONS – further details

A comparison of the delivery and composition of the two cardioplegia methods is shown below.

|  | Cold cardioplegia | Warm cardioplegia |
| --- | --- | --- |
| Further detail | Also known as Harefield 4:1 cold blood Cardioplegia | A type of microplegia |
| Preparation | 100ml (5 x 20ml ampules) of Martindale sterile concentration for Cardioplegia Infusion, and inject this into a 1litre bag or Ringers  **OR**  High Strength Cardioplegia Solution (Harefield hospital formulation), manufactured by **Terumo** BCT Ltd.* | 20 ml ampules of Martindale sterile concentration for Cardioplegia Infusion drawn up into a 50 ml syringe and inserted into a syringe driver. |
| Blood cardioplegia infusion/mix procedure | Crystalloid element is mixed with patients’ blood to a ratio of 1:4 controlled via roller pumps on the heart lung machine. | A patient’s surface area is looked up on the warm cardioplegia chart. This chart is used to determine infusion rate on the syringe driver in ml/hr. The arterial blood flow will match this in ml/min. |
| Dilution/dose | For every 1000 ml of blood cardioplegia given there will be 800 ml of patients’ blood to 200ml crystalloid cardioplegia | Much smaller dilution. The volume of the crystalloid cardioplegia is 60 times less than the blood volume. In 1000 ml of warm blood cardioplegia there will be 16.6ml of cardioplegia infusion (Martindale). |
| End point K+ concentration mmol/L | 20 plus patient’s serum K+ | 13.5 plus patient’s serum K+ |
| End point Mg2+ concentration mmol/L | 16 | 13.5 |
| Speed of delivery | Total cardioplegia volume worked out by using 110 ml/m2/min. Induction dose is 4 minutes, and subsequent doses are 2 minutes. | Blood flow in ml/min determined by body surface area and warm blood cardioplegia chart. Induction dose is 1 minute after electromechanical arrest. Subsequent doses are 1 minute following the chart dose. |
| Maintenance | After 20 – 30 mins ischaemic time | After 15 mins ischaemic time |

* Harefield hospital formulation was used initially but became unavailable, necessitating a switch to Martindale solution part-way through the study.

# Study outcomes – further details

The timing at which study outcomes were measured is shown below.

| *Data or samples collected* | *Pre-*  *surgery* | *Perioperative* | | |  |  |  |  |  |  |  |  |  |  |
| --- | --- | --- | --- | --- | --- | --- | --- | --- | --- | --- | --- | --- | --- | --- |
|  |  | Start  of  CPB | 10 min  Post  CPB | XC  removal | XC  +  1hr | XC  + 2hr | XC  +  4hr | XC  +  6hr | XC  +  12hr | XC  +  24hr | XC  +  48hr | Subsequent days up to hospital discharge | Hospital discharge | 3  months post-surgery |
| Blood gases | ***🗸*** |  |  | ***🗸*** | ***🗸*** |  | ***🗸*** |  |  | ***🗸*** | ***🗸*** |  |  |  |
| Blood samples | ***🗸*** |  |  |  |  |  | ***🗸*** |  |  |  | ***🗸*** | ***🗸*** |  |  |
| Blood for primary outcome (cTnT) | ***🗸*** |  |  |  |  | ***🗸*** |  | ***🗸*** |  | ***🗸*** | ***🗸*** |  |  |  |
| CVS |  |  |  |  | ***🗸*** | ***🗸*** | ***🗸*** | ***🗸*** | ***🗸*** | ***🗸*** | ***🗸*** |  |  |  |
| Arterial saturations | ***🗸*** |  |  |  | ***🗸*** |  | ***🗸*** |  | ***🗸*** | ***🗸*** |  |  |  |  |
| Urinary albumin and creatinine, RBP, NAG and n-GAL | ***🗸*** |  |  |  |  |  | ***🗸*** |  |  | ***🗸*** | ***🗸*** |  |  |  |
| Waste heart tissue (if available) |  |  |  | ***🗸*** |  |  |  |  |  |  |  |  |  |  |
| Operative details | ***🗸*** | ***🗸*** | ***🗸*** | ***🗸*** |  |  |  |  |  |  |  |  |  |  |
| Clinical outcomes |  |  |  |  |  |  |  |  |  |  |  |  | ***🗸*** |  |
| Safety data post discharge |  |  |  |  |  |  |  |  |  |  |  |  |  | ***🗸*** |

CPB = cardiopulmonary bypass, CVS = central venous saturations, XC = cross-clamp, cTnT = cardiac troponin T, RBP = retinol binding protein, NAG = N-acetyl-β-glucosaminidase, n-GAL = neutrophil gelatinase-associated lipocalin

Central venous saturation measurements were taken at varying time points between cross-clamp removal and 48 hours thereafter. Central arterial saturation measurements were taken at induction and up to 24 hours postoperatively. Cardiac troponin T (cTnT) and routine blood gas measurements were made pre-surgery and up to 48 hours after cross-clamp removal. Routine blood test measurements were made pre-surgery and from 4 hours after cross-clamp removal until discharge. Urine samples were taken pre-surgery and up to 48 hours after cross-clamp removal. Although the protocol allowed for analysis of waste tissue, the number of samples collected/amount available was not sufficient.

# Statistical methods – further details

Continuous data were summarised using mean and standard deviation (or median and interquartile range (IQR)/geometric mean and 95% confidence interval (CI) if distributions are skewed) and categorical data as a number and percentage. Statistical model fit was assessed via standard methods (e.g. graphical plots) and if inadequate then transformations or alternative analysis methods were sought. Some outcomes involving serial measurements taken as part of routine care were described but not formally compared to minimise the type 1 errors. These were pre-specified in the statistical analysis plan. Binary outcomes are described but not formally compared due to the low power. Three sensitivity analyses were pre-specified in the statistical analysis plan but not in the trial protocol for the primary outcome: a) excluding all protocol deviations with the exception of those relating to unblinding of non-laboratory staff; b) excluding all patients receiving one or more cardioplegia infusion outside the allocated temperature range; and c) excluding all patients receiving one or more cardioplegia infusion within the alternate temperature range to that allocated (crossovers). No sub-group analyses were planned.

For time to event outcomes (e.g. intubation time, length of postoperative hospital stay) participants who did not experience the outcome were censored at time of death (or time of transfer to another hospital for length of postoperative hospital stay)

Mixed models of longitudinal outcomes included time (category variable), treatment group, treatment by time, risk adjustment for congenital heart surgery (RACHS) strata and baseline measure fitted as fixed effects and participant and time fitted as random effects. If the interaction between treatment group and time was statistically significant at p<0.1 results are presented for each time point separately, otherwise the treatment by time interaction was removed from the model and an overall treatment effect is given. Alternative variance/covariance structures were compared (using likelihood ratio tests) to best allow for the correlation between measurements taken at different times for the same participant. Baseline measurements were fitted as a continuous covariate, except for cTnT. As a large proportion of participants had preoperative cTnT concentrations below the limit of detection (5ng/mL), baseline values were categorised as undetectable, detectable but below the median detectable value or detectable but above the median detectable value.

For central venous saturation over the first 48 hours postoperatively (where measurements were not taken at specified at fixed points in time), the minimum value recorded was determined for each participant and compared using linear regression.

The ordinal outcomes of left ventricular and right ventricular function (ranging from 0=normal to 4=severe impairment) were measured pre- and postoperatively. As preoperative function was normal for the majority of participants with data available (60/67 [90%] for left ventricular function and 58/65 [89%] for right ventricular function) and levels of missing data were relatively high (>30%), the models were not adjusted for preoperative function. Multiple imputation using chained equations (with 10 imputed datasets) was used to handle missing postoperative left ventricular and right ventricular function data (11% and 12% of data, respectively). For all other outcomes missing data levels were lower and complete case analyses were used.
