# Supplementary material for: Warm versus cold blood cardioplegia in paediatric congenital heart surgery: a randomized trial
Source: Eur J Cardiothorac Surg. 2023 Feb 17;63(4):ezad041. doi: 10.1093/ejcts/ezad041 (PMC10097434; doi:10.1093/ejcts/ezad041)
Supplement: ezad041_Supplementary_Data [file ezad041_supplementary_data.zip › Supplementary Tables S1 to S15.docx]

Supplementary Tables

Contents

[Table S 1 Protocol deviations 1](#_Toc98761475)

[Table S 2 Details of protocol deviations relating to unblinding of staff 2](#_Toc98761476)

[Table S 3 Withdrawals 3](#_Toc98761477)

[Table S 4 Patient demography and past history: further details 4](#_Toc98761478)

[Table S 5 Intraoperative characteristics 5](#_Toc98761479)

[Table S 6 Primary outcome 10](#_Toc98761480)

[Table S 7 Secondary outcomes and related data I: General ^1^ 11](#_Toc98761481)

[Table S 8 Secondary outcomes and related data II: Cardiac electrical activity 13](#_Toc98761482)

[Table S 9 Longitudinal secondary outcomes I: Cardiac function - direct measures of cardiac index 15](#_Toc98761483)

[Table S 10 Longitudinal secondary outcomes II: Cardiac function - indirect measures 16](#_Toc98761484)

[Table S 11 Longitudinal secondary outcomes III: Blood and blood gas results 17](#_Toc98761485)

[Table S 12 Secondary outcomes and related data III: Chest and wound infections 19](#_Toc98761486)

[Table S 13 Postoperative details 21](#_Toc98761487)

[Table S 14 Hospital re-admissions within 3 months of operation 24](#_Toc98761488)

[Table S 15 Details of hospital readmissions (within 3 months of operation) for reasons not listed in the protocol as expected events 25](#_Toc98761489)

Table S 1 Protocol deviations

|  |  | Randomised to ICBC (n=48) | | Randomised to IWBC (n=49) | | Overall (n=97) | |
| --- | --- | --- | --- | --- | --- | --- | --- |
| Deviation |  | n | % | n | % | n | % |
| **Any protocol deviation** |  | 7/44 | 15.9% | 10/47 | 21.3% | 17/91 | 18.7% |
| **All protocol deviations (events/patients)** |  | 12/7 | 15.9% | 17/10 | 21.3% | 29/17 | 18.7% |
| Patient received treatment other than that allocated (cardioplegia temperature not within specified range) ^1^ |  | 1/44 | 2.3% | 4/47 | 8.5% | 5/91 | 5.5% |
| Crossovers (cardioplegia temperature within alternative range)^2,3,4^ |  | 1/46 | 2.2% | 3/49 | 6.1% | 4/95 | 4.2% |
| Blinded member of staff was unblinded |  | 7/48 | 14.6% | 9/49 | 18.4% | 16/97 | 16.5% |

ICBC = intermittent cold blood cardioplegia, IWBC = intermittent warm blood cardioplegia

***Notes:***

*^1^ Patients allocated to ICBC but receiving cardioplegia that was not between 2 and 8°C, or patients allocated to IWBC but receiving cardioplegia that was < 32°C (note: a deviation of 2°C or less from the temperature range specified in the protocol is not considered as a protocol deviation)*

*^2^ Patients allocated to ICBC but receiving cardioplegia that was >20°C, or patients allocated to IWBC but receiving cardioplegia that was < 20°C*

*^3^ One patient allocated to IWBC had two cardioplegia infusions at 26°C: this was considered to be a protocol deviation but not a crossover.*

*^4^ Reasons for crossover:*

*ICBC: unrecorded.*

*IWBC: inability to arrest; error in cardioplegia delivery (32^o^C instead of >34^o^C); concern about the length of*

*procedure by the perfusionist after allocation*

Table S 2 Details of protocol deviations relating to unblinding of staff

|  |  | Randomised to ICBC (n=48) | | Randomised to IWBC (n=49) | | Overall (n=97) | |
| --- | --- | --- | --- | --- | --- | --- | --- |
| Deviation detail |  | n | % | n | % | n | % |
| Number of unblinding incidents | (events/patients) | 10/7 | 14.6% | 10/9 | 18.4% | 20/16 | 16.5% |
| Personnel unblinded | Staff nurse | 2/10 | 20.0% | 3/10 | 30.0% | 5/20 | 25.0% |
|  | Staff nurse (PICU) | 0/10 | 0.0% | 1/10 | 10.0% | 1/20 | 5.0% |
|  | Data collection nurse | 0/10 | 0.0% | 2/10 | 20.0% | 2/20 | 10.0% |
|  | Cardiac data nurse | 1/10 | 10.0% | 0/10 | 0.0% | 1/20 | 5.0% |
|  | Consultant anaesthetist | 1/10 | 10.0% | 0/10 | 0.0% | 1/20 | 5.0% |
|  | Doctor | 0/10 | 0.0% | 1/10 | 10.0% | 1/20 | 5.0% |
|  | Transfusion practitioner | 0/10 | 0.0% | 1/10 | 10.0% | 1/20 | 5.0% |
|  | Potential unblinding: PICU nursing/PICU medical/ Cardiology medical staff | 6/10 | 60.0% | 2/10 | 20.0% | 8/20 | 40.0% |
| Reason unblinded | Checking anaesthetics | 0/10 | 0.0% | 3/10 | 30.0% | 3/20 | 15.0% |
|  | Checking volume of modified ultrafiltration blood | 0/10 | 0.0% | 1/10 | 10.0% | 1/20 | 5.0% |
|  | Checking timing of medications during surgery | 1/10 | 10.0% | 0/10 | 0.0% | 1/20 | 5.0% |
|  | Checking timing of antibiotic administration | 0/10 | 0.0% | 1/10 | 10.0% | 1/20 | 5.0% |
|  | For anaesthetic alert review prior to theatre | 1/10 | 10.0% | 0/10 | 0.0% | 1/20 | 5.0% |
|  | Data collection – unspecified | 0/10 | 0.0% | 1/10 | 10.0% | 1/20 | 5.0% |
|  | Data required for audit purposes | 1/10 | 10.0% | 2/10 | 20.0% | 3/20 | 15.0% |
|  | Envelope found open and resealed | 1/10 | 10.0% | 1/10 | 10.0% | 2/20 | 10.0% |
|  | Surgeon unblinded mother who unblinded bedside nurse | 1/10 | 10.0% | 0/10 | 0.0% | 1/20 | 5.0% |
|  | Surgeon unblinded mother and she may have unblinded others besides bedside nurse | 1/10 | 10.0% | 0/10 | 0.0% | 1/20 | 5.0% |
|  | Study allocation recorded in daily/online PICU notes | 3/10 | 30.0% | 0/10 | 0.0% | 3/20 | 15.0% |
|  | Anaesthetic and/or perfusion charts not replaced in sealed envelope | 1/10 | 10.0% | 1/10 | 10.0% | 2/20 | 10.0% |

ICBC = intermittent cold blood cardioplegia, IWBC = intermittent warm blood cardioplegia, PICU = paediatric intensive care unit

Table S 3 Withdrawals

|  | | | Randomised to ICBC (n=48) | | Randomised to IWBC (n=49) | | **Overall (n=97)** | |
| --- | --- | --- | --- | --- | --- | --- | --- | --- |
| **Category** | | | **n** | **%** | **n** | **%** | **n** | **%** |
| **Any withdrawal** | |  | 2/48 | 4.2% | 0/49 | 0.0% | 2/97 | 2.1% |
| **Timing of withdrawal from study** | | |  |  |  |  |  |  |
|  | Before surgery | | 1/2 | 50.0% |  |  | 1/2 | 50.0% |
|  | During surgery | | 1/2 | 50.0% |  |  | 1/2 | 50.0% |
| **Decision to exit study taken by** | | |  |  |  |  |  |  |
|  | Parent^1^ | | 1/2 | 50.0% |  |  | 1/2 | 50.0% |
|  | Clinician^2^ | | 1/2 | 50.0% |  |  | 1/2 | 50.0% |
| **Reason for withdrawal** | | |  |  |  |  |  |  |
|  | Patient/parent has changed mind | | 1/2 | 50.0% |  |  | 1/2 | 50.0% |
|  | Patient no longer eligible | | 1/2 | 50.0% |  |  | 1/2 | 50.0% |

ICBC = intermittent cold blood cardioplegia, IWBC = intermittent warm blood cardioplegia

***Notes:***

*^1^ Mum felt she had no headspace for research after operation cancelled 4 times. Permission to keep data collected so far and to use routine data was given.*

*^2^ Patient withdrawn as they did not need to have cardioplegia during their operation. Permission to keep data collected so far was given.*

Table S 4 Patient demography and past history: further details

|  |  | Randomised to ICBC (n=48) | | Randomised to IWBC (n=49) | | Overall  (n=97) | |
| --- | --- | --- | --- | --- | --- | --- | --- |
| Characteristic |  | n | % | n | % | n | % |
| PREVIOUS CARDIAC SURGERY | | | |  |  |  |  |
| Previous cardiac operation |  | 19/48 | 39.6% | 24/49 | 49.0% | 43/97 | 44.3% |
| Oxygen saturations (%) | median (IQR) | 98 | (96.0, 99.0) | 96 | (88.0, 99.0) | 97 | (92.0, 99.0) |
| HEART RHYTHM |  |  |  |  |  |  |  |
| Sinus rhythm |  | 47/48 | 97.9% | 49/49 | 100.0% | 96/97 | 99.0% |
| Heart block |  | 1/48 | 2.1% | 0/49 | 0.0% | 1/97 | 1.0% |
| Atrial fibrillation/flutter |  | 0/48 | 0.0% | 0/49 | 0.0% | 0/97 | 0.0% |
| Paced |  | 1/48 | 2.1% | 1/49 | 2.0% | 2/97 | 2.1% |
| DRUGS ON ADMISSION |  |  |  |  |  |  |  |
| Beta blockers |  | 5/48 | 10.4% | 5/49 | 10.2% | 10/97 | 10.3% |
| Diuretics |  | 16/48 | 33.3% | 12/49 | 24.5% | 28/97 | 28.9% |
| ACE inhibitors |  | 9/48 | 18.8% | 11/49 | 22.4% | 20/97 | 20.6% |
| Sildenafil |  | 1/48 | 2.1% | 0/49 | 0.0% | 1/97 | 1.0% |
| Aspirin |  | 3/48 | 6.3% | 10/49 | 20.4% | 13/97 | 13.4% |
| Warfarin |  | 1/48 | 2.1% | 0/49 | 0.0% | 1/97 | 1.0% |
| Other |  | 17/48 | 35.4% | 18/49 | 36.7% | 35/97 | 36.1% |

ICBC = intermittent cold blood cardioplegia, IWBC = intermittent warm blood cardioplegia, IQR = interquartile range, ACE = Angiotensin-converting enzyme

Table S 5 Intraoperative characteristics

|  | | |  | Randomised to ICBC (n=48) | | Randomised to  IWBC (n=49) | | Overall (n=97) | |
| --- | --- | --- | --- | --- | --- | --- | --- | --- | --- |
| Characteristic | | |  | n | % | n | % | n | % |
| OPERATION DETAILS | | |  |  |  |  |  |  |  |
| Duration of operation (minutes) ^1^ | | | Median (IQR) | 228 | (182, 316) | 225 | (161,270) | 225 | (167, 285) |
| Alive at end of operation | | |  | 48/48 | 100.0% | 49/49 | 100.0% | 97/97 | 100.0% |
| BYPASS DATA | | |  |  |  |  |  |  |  |
| Bypass used a second time | | |  | 3/47 | 6.4% | 4/49 | 8.2% | 7/96 | 7.3% |
| Cross-clamp used a second time | | |  | 3/47 | 6.4% | 3/49 | 6.1% | 6/96 | 6.3% |
| ECMO | | |  | 0/47 | 0.0% | 0/49 | 0.0% | 0/96 | 0.0% |
| INOTROPIC SUPPORT COMING OFF BYPASS | | | | |  |  |  |  |  |
| Dopamine | | |  | 37/48 | 77.1% | 38/49 | 77.6% | 75/97 | 77.3% |
|  | Dose (µg/kg/min) | | Median (IQR) | 5.0 | (5.0, 5.0) | 5.0 | (5.0, 5.0) | 5.0 | (5.0, 5.0) |
| Milrinone | | |  | 45/48 | 93.8% | 48/49 | 98.0% | 93/97 | 95.9% |
|  | Dose (µg/kg/min) | | Median (IQR) | 0.50 | (0.25, 0.50) | 0.50 | (0.50, 0.50) | 0.50 | (0.30, 0.50) |
| Adrenaline | | |  | 5/48 | 10.4% | 7/49 | 14.3% | 12/97 | 12.4% |
|  | Dose (µg/kg/min) | | Median (IQR) | 0.05 | (0.05, 0.08) | 0.05 | (0.02, 0.10) | 0.05 | (0.03, 0.09) |
| Noradrenaline | | |  | 2/48 | 4.2% | 5/49 | 10.2% | 7/97 | 7.2% |
|  | Dose (µg/kg/min) | | Median (IQR) | 0.06 | (0.02, 0.10) | 0.10 | (0.05, 0.10) | 0.10 | (0.02, 0.10) |
| Dobutamine | | |  | 0/48 | 0.0% | 0/49 | 0.0% | 0/97 | 0.0% |
| OTHER DETAILS | | |  |  |  |  |  |  |  |
| Intra-operative insulin infusion | | |  | 0/48 | 0.0% | 0/49 | 0.0% | 0/97 | 0.0% |
| Intra-operative vasodilator | | |  | 33/48 | 68.8% | 26/49 | 53.1% | 59/97 | 60.8% |
|  | | |  |  |  |  |  |  |  |
| ANAESTHESIA | | |  |  |  |  |  |  |  |
| Sevoflurane used for induction of anaesthesia | | |  | 37/48 | 77.1% | 42/49 | 85.7% | 79/97 | 81.4% |
| If sevofluorane not used, | | | |  |  |  |  |  |  |
|  | | Reason | Intravenous induction | 3/11 | 27.3% | 5/7 | 71.4% | 8/18 | 44.4% |
|  | |  | Aortic stenosis | 1/11 | 9.1% | 0/7 | 0.0% | 1/18 | 5.6% |
|  | |  | Old child with multiple sclerosis | 1/11 | 9.1% | 0/7 | 0.0% | 1/18 | 5.6% |
|  | |  | Critical atrial fibrillation | 1/11 | 9.1% | 0/7 | 0.0% | 1/18 | 5.6% |
|  | |  | Propofol induction and maintenance | 1/11 | 9.1% | 0/7 | 0.0% | 1/18 | 5.6% |
|  | |  | Other | 0/11 | 0.0% | 1/7 | 14.3% | 1/18 | 5.6% |
|  | |  | Unknown | 4/11 | 36.4% | 1/7 | 14.3% | 5/18 | 27.8% |
|  | | Anaesthetic used instead | Ketamine | 3/11 | 27.3% | 3/7 | 42.9% | 6/18 | 33.3% |
|  | |  | Propofol | 4/11 | 36.4% | 3/7 | 42.9% | 7/18 | 38.9% |
|  | |  | Midazolam | 1/11 | 9.1% | 0/7 | 0.0% | 1/18 | 5.6% |
|  | |  | Propofol and Fentanyl | 1/11 | 9.1% | 0/7 | 0.0% | 1/18 | 5.6% |
|  | |  | Ketamine and Propofol | 1/11 | 9.1% | 0/7 | 0.0% | 1/18 | 5.6% |
|  | |  | Fentanyl and Ketamine | 1/11 | 9.1% | 0/7 | 0.0% | 1/18 | 5.6% |
|  | |  | Isoflurane | 0/11 | 0.0% | 1/7 | 14.3% | 1/18 | 5.6% |
| Pancuronium used for non-depolarising muscle relaxant | | |  | 30/48 | 62.5% | 31/49 | 63.3% | 61/97 | 62.9% |
| If pancuronium not used, | | |  |  |  |  |  |  |  |
|  | | Reason | Fast track | 7/18 | 38.9% | 10/18 | 55.6% | 17/36 | 47.2% |
|  | |  | Other | 7/18 | 38.9% | 3/18 | 16.7% | 10/36 | 27.8% |
|  | |  | Unknown | 4/18 | 22.2% | 5/18 | 27.8% | 9/36 | 25.0% |
|  | | Relaxant used instead | Vecuronium | 1/18 | 5.6% | 1/18 | 5.6% | 2/36 | 5.6% |
|  | |  | Rocuronium | 6/18 | 33.3% | 9/18 | 50.0% | 15/36 | 41.7% |
|  | |  | Atracurium | 10/18 | 55.6% | 7/18 | 38.9% | 17/36 | 47.2% |
|  | |  | Not known | 1/18 | 5.6% | 1/18 | 5.6% | 2/36 | 5.6% |
| Fentanyl (5-20mcg/kg) given prior to CPB | | |  | 45/48 | 93.8% | 49/49 | 100.0% | 94/97 | 96.9% |
| If fentanyl used, | | |  |  |  |  |  |  |  |
|  | | Dose ^2^ (mcg/kg) | Mean (SD) | 13 | 5.1 | 14 | 6.7 | 13 | 6.0 |
| If fentanyl not used ^3^, | | |  |  |  |  |  |  |  |
|  | | Reason | Remifentanil used | 2/3 | 66.7% |  |  | 2/3 | 66.7% |
|  | |  | Unknown | 1/3 | 33.3% |  |  | 1/3 | 33.3% |
|  | |  |  |  |  |  |  |  |  |
| Maintenance of anaesthesia prior to CPB | | | Isoflurane | 43/48 | 89.6% | 44/49 | 89.8% | 87/97 | 89.7% |
|  |  |  | Propofol | 3/48 | 6.3% | 0/49 | 0.0% | 3/97 | 3.1% |
|  |  |  | Other ^3^ | 2/48 | 4.2% | 5/49 | 10.2% | 7/97 | 7.2% |
|  |  |  |  |  |  |  |  |  |  |
| If other, | | |  |  |  |  |  |  |  |
|  | | Drug used | Sevoflurane | 1/2 | 50.0% | 2/5 | 40.0% | 3/7 | 42.9% |
|  | |  | Ketamine | 0/2 | 0.0% | 1/5 | 20.0% | 1/7 | 14.3% |
|  | |  | Sevoflurane and midazolam | 1/2 | 50.0% | 1/5 | 20.0% | 2/7 | 28.6% |
|  | |  | Isoflurane and ketamine | 0/2 | 0.0% | 1/5 | 20.0% | 1/7 | 14.3% |
|  | |  |  |  |  |  |  |  |  |
|  | | Reason | Regurgitation | 0/2 | 0.0% | 1/5 | 20.0% | 1/7 | 14.3% |
|  | |  | Stenosis | 1/2 | 50.0% | 1/5 | 20.0% | 2/7 | 28.6% |
|  | |  | Less systemic vascular resistance | 1/2 | 50.0% | 0/5 | 0.0% | 1/7 | 14.3% |
|  | |  | Reason missing | 0/2 | 0.0% | 3/5 | 60.0% | 3/7 | 42.9% |
| Maintenance of anaesthesia during CPB | | | Isoflurane | 34/48 | 70.8% | 33/49 | 67.3% | 67/97 | 69.1% |
|  |  |  | Propofol | 9/48 | 18.8% | 13/49 | 26.5% | 22/97 | 22.7% |
|  |  |  | Other ^3^ | 5/48 | 10.4% | 3/49 | 6.1% | 8/97 | 8.2% |
| If other, | | |  |  |  |  |  |  |  |
|  | | Drug used | Ketamine | 1/5 | 20.0% | 0/3 | 0.0% | 1/8 | 12.5% |
|  | | | Midazolam, morphine and isoflurane | 1/5 | 20.0% | 1/3 | 33.3% | 2/8 | 25.0% |
|  | | | Isoflurane and ketamine | 0/5 | 0.0% | 1/3 | 33.3% | 1/8 | 12.5% |
|  | | | Isoflurane and propofol | 1/5 | 20.0% | 0/3 | 0.0% | 1/8 | 12.5% |
|  | | | Propofol and remifentanil | 1/5 | 20.0% | 0/3 | 0.0% | 1/8 | 12.5% |
|  | | | Unknown | 1/5 | 20.0% | 1/3 | 33.3% | 2/8 | 25.0% |
|  | | |  |  |  |  |  |  |  |
|  | | Reason | Stenosis | 0/5 | 0.0% | 1/3 | 33.3% | 1/8 | 12.5% |
|  | |  | TIVA | 2/5 | 40.0% | 0/3 | 0.0% | 2/8 | 25.0% |
|  | |  | Isoflurane not reliable as sole agent | 0/5 | 0.0% | 1/3 | 33.3% | 1/8 | 12.5% |
|  | |  | Isoflurane and propofol given | 1/5 | 20.0% | 0/3 | 0.0% | 1/8 | 12.5% |
|  | |  | Reason missing | 2/5 | 40.0% | 1/3 | 33.3% | 3/8 | 37.5% |
|  | |  |  |  |  |  |  |  |  |
| Maintenance of anaesthesia after CPB | | | Isoflurane | 39/48 | 81.3% | 43/49 | 87.8% | 82/97 | 84.5% |
|  |  |  | Propofol | 6/48 | 12.5% | 1/49 | 2.0% | 7/97 | 7.2% |
|  |  |  | Other ^3^ | 3/48 | 6.3% | 5/49 | 10.2% | 8/97 | 8.2% |
| If other, | | |  |  |  |  |  |  |  |
|  | | Drug used | Isoflurane and propofol | 1/3 | 33.3% | 0/5 | 0.0% | 1/8 | 12.5% |
|  | |  | Sevoflurane | 0/3 | 0.0% | 2/5 | 40.0% | 2/8 | 25.0% |
|  | |  | Midazolam, morphine and isoflurane | 1/3 | 33.3% | 1/5 | 20.0% | 2/8 | 25.0% |
|  | |  | Propofol and sevoflurane | 0/3 | 0.0% | 1/5 | 20.0% | 1/8 | 12.5% |
|  | |  | Unknown | 1/3 | 33.3% | 1/5 | 20.0% | 2/8 | 25.0% |
|  | |  |  |  |  |  |  |  |  |
|  | | Reason | To prevent awareness and give analgesia | 1/3 | 33.3% | 0/2 | 0.0% | 1/5 | 20.0% |
|  | |  | Usual pre-PICU drugs | 0/3 | 0.0% | 1/2 | 50.0% | 1/5 | 20.0% |
|  | |  | Unknown | 2/3 | 66.7% | 1/2 | 50.0% | 3/5 | 60.0% |
| BLOOD MANAGEMENT TECHNIQUES | | |  |  |  |  |  |  |  |
| Tranexamic acid | | |  | 36/48 | 75.0% | 36/49 | 73.5% | 72/97 | 74.2% |
| Dose of tranexamic acid (g) ^4^ | | | median (IQR) | 0.2 | (0.20, 0.63) | 0.4 | (0.18, 0.51) | 0.3 | (0.20, 0.57) |
| Aprotinin | | |  | 5/48 | 10.4% | 8/49 | 16.3% | 13/97 | 13.4% |
| Dose of aprotinin (1,000 units) | | | median (IQR) | 338 | (300, 680) | 241250 | (828, 1567500) | 960 | (338, 480000) |
| BLOOD PRODUCTS | | |  |  |  |  |  |  |  |
| RBC | | |  | 26/48 | 54.2% | 27/49 | 55.1% | 53/97 | 54.6% |
| Total intra-operative  RBC (mL) | | | median (IQR) | 65 | (50.0, 180.0) | 100 | (100.0, 175.0) | 100 | (60.0, 175.0) |
| FFP | | |  | 2/48 | 4.2% | 1/49 | 2.0% | 3/97 | 3.1% |
| Total intra-operative  FFP (mL) | | | median (IQR) | 240 | (80.0, 400.0) | 400 | (400.0, 400.0) | 400 | (80.0, 400.0) |
| Platelets | | |  | 23/47 | 48.9% | 32/49 | 65.3% | 55/96 | 57.3% |
| Total intra-operative  platelets (mL) | | | median (IQR) | 75 | (50.0, 150.0) | 105 | (55.0, 180.0) | 100 | (50.0, 160.0) |
| Cryoprecipitate | | |  | 12/47 | 25.5% | 20/49 | 40.8% | 32/96 | 33.3% |
| Total intra-operative  cryoprecipitate (mL) | | | median (IQR) | 50 | (40.0, 90.0) | 51 | (40.0, 100.0) | 50 | (40.0, 100.0) |
| NIRS | | |  |  |  |  |  |  |  |
| NIRS (%) | | |  |  |  |  |  |  |  |
|  | pre-CPB | | median (IQR) | 79 | (69.5, 88.0) | 77 | (67.0, 86.0) | 78 | (68.0, 87.0) |
|  | 1st record post start CPB | | median (IQR) | 68 | (60.0, 74.5) | 66 | (58.0, 75.0) | 67 | (60.0, 75.0) |
|  | start CPB+30 min ^4^ | | median (IQR) | 71 | (63.0, 77.0) | 69 | (64.0, 73.0) | 69 | (64.0, 75.5) |
|  | start CPB+60 min ^5^ | | median (IQR) | 69 | (63.0, 75.0) | 66 | (60.0, 74.0) | 68 | (62.0, 74.0) |
|  | 1st record post end CPB ^4^ | | median (IQR) | 75 | (65.5, 83.0) | 74 | (66.0, 82.0) | 74 | (66.0, 83.0) |
|  | end CPB+15 min ^6^ | | median (IQR) | 77 | (68.0, 84.0) | 77 | (65.0, 82.0) | 77 | (66.5, 83.0) |
|  | end CPB+30 min ^7^ | | median (IQR) | 78 | (71.0, 86.0) | 77 | (69.0, 86.0) | 77 | (69.0, 86.0) |
|  | end CPB+60 min ^8^ | | median (IQR) | 76 | (69.0, 83.5) | 76 | (68.0, 84.0) | 76 | (68.0, 83.5) |
| TEMPERATURE DURING SURGERY | | | |  |  |  |  |  |  |
| Median core temperature (*°*C) ^9^ | | | median (IQR) | 35.1 | (34.3, 35.9) | 35.3 | (34.5, 35.8) | 35.3 | (34.5, 35.9) |
| Median skin temperature (*°*C) ^10^ | | | median (IQR) | 33.6 | (32.6, 34.6) | 33.6 | (32.4, 34.8) | 33.6 | (32.5, 34.8) |
| Median blood temperature (*°*C) ^11^ | | | median (IQR) | 33.9 | (32.2, 34.4) | 34.3 | (33.2, 35.6) | 34.0 | (32.4, 35.3) |

ICBC = intermittent cold blood cardioplegia, IWBC = intermittent warm blood cardioplegia, CPB = cardiopulmonary bypass, IQR = interquartile range, SD = standard deviation, ECMO = extracorporeal membrane oxygenation, TIVA = Total intravenous anaesthesia, PICU = paediatric intensive care unit, RBC = red blood cells, FFP = fresh frozen plasma, NIRS = near-infrared spectroscopy.

**Note:**

*^1^* Data were missing for 4 patients (allocated to ICBC)

^2^ 9 patients (3 randomised to ICBC, 6 randomised to IWBC) received a dose of fentanyl outside the 5-20mcg/kg range specified in the protocol anaesthesia guidelines

^3^ This was outwith the anaesthesia guidelines in the protocol

*^4^* Data were missing for 1 patient (allocated to IWBC)

*^5^* Data were missing for 16 patients (5 allocated to ICBC, 11 allocated to IWBC)

*^6^* Data were missing for 5 patients (2 allocated to ICBC, 3 allocated to IWBC)

*^7^* Data were missing for 10 patients (6 allocated to ICBC, 4 allocated to IWBC)

*^8^* Data were missing for 37 patients (16 allocated to ICBC, 21 allocated to IWBC)

*^9^* Data were missing for 6 patients (4 allocated to ICBC, 2 allocated to IWBC)

*^10^* Data were missing for 19 patients (11 allocated to ICBC, 8 allocated to IWBC)

*^11^* Data were missing for 26 patients (11 allocated to ICBC, 15 allocated to IWBC)

Table S 6 Primary outcome

| **Primary outcome** | | | Randomised to ICBC (n=48) | | Randomised to IWBC (n=49) | | Effect  (95% CI) | p-value |
| --- | --- | --- | --- | --- | --- | --- | --- | --- |
|  |  |  | median | IQR | median | IQR |  |  |
| **Cardiac Troponin T (ng/L)** | | | |  |  |  |  |  |
| Pre-surgery | |  |  |  |  |  |  |  |
|  | <5ng/L^1^ | n (%) | 13/45 | 28.9% | 10/49 | 20.4% |  |  |
|  | 5-12ng/L | n (%) | 12/45 | 26.7% | 23/49 | 46.9% |  |  |
|  | >=13nl/L | n (%) | 20/45 | 44.4% | 16/49 | 32.7% |  |  |
| Pre-surgery (>=5ng/L only) | | | 14 | (9.0, 25.5) | 10 | (6.0, 24.0) |  |  |
| *XC+2h* ^2^ | | | 2144 | (1398.5, 4658.5) | 3045 | (827.0, 6358.0) |  |  |
| *XC+6h* ^3^ | | | 1804 | (1078.0, 3730.0) | 1860 | (829.0, 4455.0) |  |  |
| *XC+24h* ^4^ | | | 1069 | (477.0, 1902.0) | 1018 | (454.5, 2393.0) |  |  |
| *XC+48h* ^5^ | | | 869 | (386.0, 1500.0) | 1022 | (372.0, 1835.0) |  |  |
| Test for treatment*time interaction | | |  |  |  |  |  | 0.93 |
| Overall treatment effect estimate^6^ | | |  |  |  |  | GMR=1.07 (0.79,1.44) | 0.66 |

ICBC = intermittent cold blood cardioplegia, IWBC = intermittent cold blood cardioplegia, IQR = interquartile range, CI = confidence interval, XC = cross-clamp, GMR = geometric mean ratio

**Note:**

Data from 95 patients (46 allocated to ICBC, 49 allocated to IWBC) contributed to the regression analysis (the missing baseline troponin category was imputed for 1 patient (ICBC) that had postoperative troponin data recorded).

^1^ the assay lower detection limit is 5ng/mL

^2^ Data were missing for 6 patients (4 allocated to ICBC, 2 allocated to IWBC)

^3^ Data were missing for 7 patients (5 allocated to ICBC, 2 allocated to IWBC)

^4^ Data were missing for 3 patients (2 allocated to ICBC, 1 allocated to IWBC)

^5^ Data were missing for 17 patients (7 allocated to ICBC, 10 allocated to IWBC)

^6^ Two sensitivity analyses were carried out. The first excluded all protocol deviations with the exception of those relating to unblinding of non-laboratory staff (n=90: 45 ICBC 45 IWBC, GMR=0.99 (0.73,1.34), p=0.94) and the second excluded all patients receiving one or more cardioplegia infusion within the alternative range to that allocated (crossovers) (n=91: 45 ICBC 46 IWBC, GMR=0.99 (0.73,1.34), p=0.94).

Table S 7 Secondary outcomes and related data I: General ^1^

|  | |  | Randomised to ICBC (n=48) | | Randomised to IWBC (n=49) | | Effect  (95% CI) | p-value |
| --- | --- | --- | --- | --- | --- | --- | --- | --- |
| **Outcome (or related data)** | |  | median | IQR | median | IQR |  |  |
| POSTOPERATIVE BLOOD LOSS | | |  |  |  |  |  |  |
| Total chest drain loss in the first 12 hours (mL) | |  | 75 | (45.0, 110.0) | 75 | (50.0, 105.0) | GMR=1.04 (0.82,1.32) | 0.73 |
| VASOACTIVE INOTROPE SCORE (VIS) | | | |  |  |  |  |  |
| Mean VIS | 0-24 hours |  | 7 | (4.9, 9.5) | 8 | (5.0, 10.0) | GMR=0.96 (0.71,1.30) | 0.78 |
|  | 24-48 hours |  | 1 | (0.0, 5.0) | 1 | (0.0, 7.2) | GMR=1.06 (0.53,2.11) | 0.87 |
|  | 0-48 hours |  | 5 | (2.8, 6.4) | 5 | (2.6, 9.2) | GMR=0.97 (0.67,1.40) | 0.87 |
| Maximum VIS | 0-24 hours |  | 10 | (6.7, 10.0) | 10 | (10.0, 12.0) |  |  |
|  | 24-48 hours |  | 5 | (0.0, 6.7) | 5 | (0.0, 10.0) |  |  |
|  | 0-48 hours |  | 10 | (6.7, 10.0) | 10 | (10.0, 12.0) |  |  |
| LENGTH OF PICU STAY | | |  |  |  |  |  |  |
| Time from return from theatre until discharge to ward/HDU (hours^) 2,3^ | |  | 45.8 | (25.6, 54.1) | 60.4 | (28.5, 122.6) | HR=0.52 (0.34,0.79) | 0.003 |
| Discharge destination | |  |  |  |  |  |  |  |
|  | Level 2 care (e.g. HDU) | n, (%) | 37/47 | 78.7% | 33/49 | 67.3% |  |  |
|  | Level 0/1 care (e.g. general ward) | n, (%) | 10/47 | 21.3% | 15/49 | 30.6% |  |  |
|  | Hospital discharge home | n, (%) | 0/47 | 0.0% | 1/49 | 2.0% |  |  |
| Readmitted to PICU | | n, (%) | 3/47 | 6.4% | 5/49 | 10.2% |  |  |
| LENGTH OF POSTOPERATIVE HOSPITAL STAY | |  |  |  |  |  |  |  |
| Time from operation to hospital discharge (hours) | |  | 160.5 | (120.5, 222.6) | 192.0 | (124.0, 314.5) | HR=0.66 (0.43,1.02) | 0.060 |
| Discharge destination | | n, (%) |  |  |  |  |  |  |
|  | Home |  | 45/47 | 95.7% | 46/49 | 93.9% |  |  |
|  | Another hospital |  | 2/47 | 4.3% | 3/49 | 6.1% |  |  |
| INTUBATION TIME | |  |  |  |  |  |  |  |
| Duration of first intubation (min) ^4^ | |  | 924 | (280.0, 1557.0) | 950 | (228.0, 3990.0) | HR=0.75 (0.50,1.14) | 0.18 |
| Reintubated | | n, (%) | 3/47 | 6.4% | 7/49 | 14.3% |  |  |
| MORTALITY | |  |  |  |  |  |  |  |
| All-cause mortality to 3 months post-surgery | | n, (%) | 0/44 | 0.0% | 0/48 | 0.0% |  |  |

ICBC = intermittent cold blood cardioplegia, IWBC = intermittent cold blood cardioplegia, IQR = interquartile range, CI = confidence interval, VIS = vasoactive inotrope score, PICU = paediatric intensive care unit, HDU = high dependency unit, GMR = geometric mean ratio, HR = hazard ratio

**Note:**

^1^ Unless otherwise stated, data were missing for 1 patient (allocated to ICBC).

^2^ Not including re-admissions to level 3 care. Although the original intention was to record timing of fitness for PICU discharge, these data were not recorded reliably so actual transfer times were used.

^3^ Data were missing for 2 patients (1 allocated to ICBC, 1 allocated to IWBC)

^4^ Data were missing for 3 patients (1 allocated to ICBC, 2 allocated to IWBC)

Table S 8 Secondary outcomes and related data II: Cardiac electrical activity

| **Outcome (or related data)** | |  | **Randomised to ICBC (n=48)** | | **Randomised to IWBC (n=49)** | | **Overall**  **(n=97)** | |
| --- | --- | --- | --- | --- | --- | --- | --- | --- |
|  |  |  | **n** | **%** | **n** | **%** | **n** | **%** |
| ARRHYTHMIAS DURING OPERATION | | | | |  |  |  |  |
| **Any arrhythmia on removal of cross clamp** | |  | 15/48 | 31.3% | 21/49 | 42.9% | 36/97 | 37.1% |
| Total arrhythmias on removal of cross clamp | | (events/ patients) | 20/15 | 31.3% | 23/21 | 42.9% | 43/36 | 37.1% |
| Type of activity: | |  |  |  |  |  |  |  |
|  | Nodal |  | 4/48 | 8.3% | 3/49 | 6.1% | 7/97 | 7.2% |
|  | Junctional ectopic tachycardia |  | 0/48 | 0.0% | 0/49 | 0.0% | 0/97 | 0.0% |
|  | Atrial fibrillation |  | 0/48 | 0.0% | 0/49 | 0.0% | 0/97 | 0.0% |
|  | Ventricular tachycardia |  | 2/48 | 4.2% | 1/49 | 2.0% | 3/97 | 3.1% |
|  | Ventricular fibrillation |  | 1/48 | 2.1% | 1/49 | 2.0% | 2/97 | 2.1% |
|  | Transient atrioventricular block, no pacing required |  | 4/48 | 8.3% | 6/49 | 12.2% | 10/97 | 10.3% |
|  | Atrioventricular block, requiring pacing in operating room |  | 3/48 | 6.3% | 8/49 | 16.3% | 11/97 | 11.3% |
|  | Atrioventricular block, requiring pacing which continues onto PICU |  | 6/48 | 12.5% | 4/49 | 8.2% | 10/97 | 10.3% |
| **Any arrhythmia on chest closure** | |  | 10/48 | 20.8% | 10/49 | 20.4% | 20/97 | 20.6% |
| Total arrhythmias on chest closure | | (events/ patients) | 11/10 | 20.8% | 10/10 | 20.4% | 21/20 | 20.6% |
| Type of activity | |  |  |  |  |  |  |  |
|  | Nodal |  | 3/48 | 6.3% | 3/49 | 6.1% | 6/97 | 6.2% |
|  | Junctional ectopic tachycardia |  | 0/48 | 0.0% | 0/49 | 0.0% | 0/97 | 0.0% |
|  | Atrial fibrillation |  | 0/48 | 0.0% | 0/49 | 0.0% | 0/97 | 0.0% |
|  | Ventricular tachycardia |  | 0/48 | 0.0% | 0/49 | 0.0% | 0/97 | 0.0% |
|  | Ventricular fibrillation |  | 0/48 | 0.0% | 0/49 | 0.0% | 0/97 | 0.0% |
|  | Transient atrioventricular block, no pacing required |  | 0/48 | 0.0% | 0/49 | 0.0% | 0/97 | 0.0% |
|  | Atrioventricular block, requiring pacing in operating room |  | 1/48 | 2.1% | 1/49 | 2.0% | 2/97 | 2.1% |
|  | Atrioventricular block, requiring pacing which continues onto PICU |  | 7/48 | 14.6% | 6/49 | 12.2% | 13/97 | 13.4% |
| If pacing was required due to atrioventricular block ^1^: | |  |  |  |  |  |  |  |
|  | Single chamber |  | 1/9 | 11.1% | 1/13 | 7.7% | 2/22 | 9.1% |
|  | Dual chamber |  | 8/9 | 88.9% | 12/13 | 92.3% | 20/22 | 90.9% |
| NEW ONSET OF ARRHYTHMIA POSTOPERATIVELY | | | | | | | | |
| **Any new onset of postoperative arrhythmia** | |  | 7/47 | 14.9% | 12/49 | 24.5% | 19/96 | 19.8% |
| Total new onsets of postoperative arrhythmia | | (events/ patients) | 8/7 | 14.9% | 15/12 | 24.5% | 23/19 | 19.8% |
| Type of arrhythmia: | |  |  |  |  |  |  |  |
|  | Supraventricular tachycardia |  | 0/47 | 0.0% | 0/49 | 0.0% | 0/96 | 0.0% |
|  | Atrial fibrillation |  | 0/47 | 0.0% | 0/49 | 0.0% | 0/96 | 0.0% |
|  | Ventricular fibrillation |  | 1/47 | 2.1% | 0/49 | 0.0% | 1/96 | 1.0% |
|  | Ventricular tachycardia |  | 0/47 | 0.0% | 1/49 | 2.0% | 1/96 | 1.0% |
|  | Heart block |  | 4/47 | 8.5% | 9/49 | 18.4% | 13/96 | 13.5% |
|  | Junctional ectopic tachycardia |  | 3/47 | 6.4% | 5/49 | 10.2% | 8/96 | 8.3% |

ICBC = intermittent cold blood cardioplegia, IWBC = intermittent cold blood cardioplegia,

**Note:**

Secondary outcomes are indicated in bold.

^1^ Data were missing for 1 patient (allocated to IWBC)

Table S 9 Longitudinal secondary outcomes I: Cardiac function - direct measures of cardiac index

|  |  | Randomised to ICBC (n=48) | | Randomised to IWBC (n=49) | | Effect^1^  (95% CI) | p-value |
| --- | --- | --- | --- | --- | --- | --- | --- |
| Outcome (or related data) | | n | % | n | % |  |  |
| **Left ventricular function** | |  |  |  |  |  |  |
| *Preoperative* | |  |  |  |  |  |  |
|  | 0 (normal) | 27/33 | 81.8% | 33/34 | 97.1% |  |  |
|  | 1 (trivial impairment) | 1/33 | 3.0% | 0/34 | 0.0% |  |  |
|  | 2 (mild impairment) | 4/33 | 12.1% | 1/34 | 2.9% |  |  |
|  | 3 (moderate impairment) | 1/33 | 3.0% | 0/34 | 0.0% |  |  |
|  | 4 (severe impairment) | 0/33 | 0.0% | 0/34 | 0.0% |  |  |
| *Postoperative* | |  |  |  |  |  |  |
|  | 0 (normal) | 34/44 | 77.3% | 32/42 | 76.2% |  |  |
|  | 1 (trivial impairment) | 0/44 | 0.0% | 3/42 | 7.1% |  |  |
|  | 2 (mild impairment) | 9/44 | 20.5% | 7/42 | 16.7% |  |  |
|  | 3 (moderate impairment) | 1/44 | 2.3% | 0/42 | 0.0% |  |  |
|  | 4 (severe impairment) | 0/44 | 0.0% | 0/42 | 0.0% |  |  |
| *Overall* | |  |  |  |  | OR=1.10 (0.42,2.84) | 0.85 |
| **Right ventricular function** | |  |  |  |  |  |  |
| *Preoperative* | |  |  |  |  |  |  |
|  | 0 (normal) | 27/31 | 87.1% | 31/34 | 91.2% |  |  |
|  | 1 (trivial impairment) | 2/31 | 6.5% | 1/34 | 2.9% |  |  |
|  | 2 (mild impairment) | 2/31 | 6.5% | 1/34 | 2.9% |  |  |
|  | 3 (moderate impairment) | 0/31 | 0.0% | 1/34 | 2.9% |  |  |
|  | 4 (severe impairment) | 0/31 | 0.0% | 0/34 | 0.0% |  |  |
| *Postoperative* | |  |  |  |  |  |  |
|  | 0 (normal) | 28/41 | 68.3% | 28/44 | 63.6% |  |  |
|  | 1 (trivial impairment) | 1/41 | 2.4% | 4/44 | 9.1% |  |  |
|  | 2 (mild impairment) | 11/41 | 26.8% | 9/44 | 20.5% |  |  |
|  | 3 (moderate impairment) | 1/41 | 2.4% | 3/44 | 6.8% |  |  |
|  | 4 (severe impairment) | 0/41 | 0.0% | 0/44 | 0.0% |  |  |
| *Overall* | |  |  |  |  | OR=1.24 (0.51,3.01) | 0.63 |
| Time from surgery to echocardiogram scan (days) ^2^ | Median (IQR) | 6 | (4.0, 9.0) | 6 | (4.5,  12.0) | 6 | (4.0,  11.0) |

ICBC = intermittent cold blood cardioplegia, IWBC = intermittent cold blood cardioplegia, CI = confidence interval, OR = odds ratio, IQR = interquartile range

***Notes:***

^1^ The odds ratios were obtained from logistic regression of postoperative function data. Adjustment for preoperative values was not carried out in this instance due to large amounts of missing data and low numbers of patients in categories other than ’normal’. Missing postoperative data were handled using a multiple imputation by chained equations approach.

^2^ Data were missing for 1 patient (allocated to IWBC)

Table S 10 Longitudinal secondary outcomes II: Cardiac function - indirect measures

|  |  | Randomised to ICBC (n=48) | | Randomised to IWBC (n=49) | | Effect  (95% CI) | p-value |
| --- | --- | --- | --- | --- | --- | --- | --- |
| **Outcome** |  | Median | (IQR) | Median | (IQR) |  |  |
| **Minimum central venous saturation (%) ^1,2^** | Mean (SD) | 65.5 | 11.3 | 62.5 | 12.4 | MD=-3.2 (-8.1,1.7) | 0.19 |
| **Central arterial saturation (%)** |  |  |  |  |  |  |  |
| *Induction* **^3^** |  | 99.5 | (97.7, 99.9) | 98.8 | (87.1, 99.5) |  |  |
| *XC + 1h* **^4^** |  | 99.5 | (98.3, 100.0) | 99.2 | (98.3, 99.8) |  |  |
| *XC + 4h* **^3^** |  | 99.8 | (99.1, 100.4) | 99.4 | (97.5, 99.8) |  |  |
| *XC + 12h* **^2^** |  | 99.1 | (98.2, 99.5) | 98.4 | (97.6, 99.3) |  |  |
| *XC + 24h* **^5^** |  | 98.1 | (96.8, 99.0) | 98.2 | (95.8, 99.1) |  |  |

ICBC = intermittent cold blood cardioplegia, IWBC = intermittent cold blood cardioplegia, IQR = interquartile range, CI = confidence interval, SD = standard deviation, MD = mean difference

***Notes:***

^1^ Between 0 and 24 hours after cross-clamp removal

^2^ Data were missing for 4 patients (3 allocated to ICBC, 1 allocated to IWBC)

^3^ Data were missing for 1 patient (allocated to ICBC)

^4^ Data were missing for 2 patients (1 allocated to ICBC, 1 allocated to IWBC)

^5^ Data were missing for 14 patients (7 allocated to ICBC, 7 allocated to IWBC)

Table S 11 Longitudinal secondary outcomes III: Blood and blood gas results

|  | | Randomised to ICBC (n=48) | | Randomised to IWBC (n=49) | | Effect  **(95% CI)** | **p-value** |  |  |
| --- | --- | --- | --- | --- | --- | --- | --- | --- | --- |
| **Outcome** | | **median** | **IQR** | **Median** | **IQR** |  |  |  |  |
| **Blood gas: lactate (mmol/L)** | |  |  |  |  |  |  |  |  |
| *Pre-surgery* ^1^ |  | 0.90 | (0.7, 1.1) | 0.80 | (0.6, 1.0) |  |  |  |  |
| *XC removal* ^2^ |  | 1.40 | (1.1, 1.9) | 1.50 | (1.3, 2.1) |  |  |  |  |
| *XC removal + 1h* ^3^ |  | 1.20 | (1.0, 2.2) | 1.20 | (1.0, 2.0) |  |  |  |  |
| *XC removal + 4h* ^4^ |  | 1.10 | (0.9, 1.7) | 1.10 | (0.9, 1.6) |  |  |  |  |
| *XC removal + 12h* ^5^ |  | 1.20 | (0.9, 1.5) | 1.10 | (0.9, 1.5) |  |  |  |  |
| *XC removal + 24h* ^6^ |  | 1.10 | (0.8, 1.4) | 1.10 | (0.9, 1.5) |  |  |  |  |
| *XC removal + 48h* ^7^ |  | 1.00 | (0.9, 1.3) | 0.90 | (0.7, 1.1) |  |  |  |  |
| Test for treatment*time interaction | |  |  |  |  |  | 0.15 |  |  |
| Overall treatment effect estimate* | |  |  |  |  | GMR=1.05 (0.95,1.15) | 0.35 |  |  |
| **Blood gas: base excess (mmol/L)** | |  |  |  |  |  |  |  |  |
| *Pre-surgery* ^4^ | Mean (SD) | -1.5 | 4.9 | -1.2 | 5.4 |  |  |  |  |
| *XC removal* ^2^ | Mean (SD) | -4.5 | 2.9 | -4.3 | 3.1 |  |  |  |  |
| *XC removal + 1h* ^3^ | Mean (SD) | -4.6 | 3.1 | -4.1 | 3.4 |  |  |  |  |
| *XC removal + 4h* ^2^ | Mean (SD) | -3.1 | 3.3 | -3.3 | 3.1 |  |  |  |  |
| *XC removal + 12h* ^8^ | Mean (SD) | -3.1 | 4.3 | -2.5 | 3.7 |  |  |  |  |
| *XC removal + 24h* ^9^ | Mean (SD) | -0.8 | 3.8 | -0.9 | 3.2 |  |  |  |  |
| *XC removal + 48h* ^10^ | Mean (SD) | 1.5 | 4.0 | 1.3 | 4.7 |  |  |  |  |
| Test for treatment*time interaction | |  |  |  |  |  | 0.81 |  |  |
| Overall treatment effect estimate** | |  |  |  |  | MD=0.016 (-0.92,0.95) | 0.97 |  |  |
| **Blood gas: pH** | |  |  |  |  |  |  |  |  |
| *Pre-surgery* ^4^ | Mean (SD) | 7.37 | 0.13 | 7.32 | 0.09 |  |  |  |  |
| *XC removal* ^4^ | Mean (SD) | 7.35 | 0.08 | 7.34 | 0.06 |  |  |  |  |
| *XC removal + 1h* ^2^ | Mean (SD) | 7.32 | 0.11 | 7.31 | 0.08 |  |  |  |  |
| *XC removal + 4h* ^4^ | Mean (SD) | 7.34 | 0.05 | 7.33 | 0.06 |  |  |  |  |
| *XC removal + 12h* ^5^ | Mean (SD) | 7.37 | 0.04 | 7.37 | 0.05 |  |  |  |  |
| *XC removal + 24h* ^6^ | Mean (SD) | 7.38 | 0.05 | 7.38 | 0.06 |  |  |  |  |
| *XC removal + 48h* ^11^ | Mean (SD) | 7.41 | 0.05 | 7.40 | 0.07 |  |  |  |  |
| Test for treatment*time interaction | |  |  |  |  |  | 0.88 |  |  |
| Overall treatment effect estimate*** | |  |  |  |  | MD=-0.005 (-0.02,0.01) | 0.53 |  |  |

ICBC = intermittent cold blood cardioplegia, IWBC = intermittent cold blood cardioplegia, IQR = interquartile range, CI = confidence interval, XC = cross-clamp, GMR = geometric mean ratio, SD = standard deviation, MD = mean difference

***Notes:***

** Data from 94 patients contributed to regression analysis (*46 allocated to ICBC, 48 allocated to IWBC)

*** Data from 95 patients contributed to regression analysis (*47 allocated to ICBC, 48 allocated to IWBC)

**** Data from 96 patients contributed to regression analysis (*47 allocated to ICBC, 49 allocated to IWBC)

^1^ Data were missing for 3 patients (2 allocated to ICBC, 1 allocated to IWBC)

^2^ Data were missing for 2 patients (1 allocated to ICBC, 1 allocated to IWBC)

^3^ Data were missing for 3 patients (1 allocated to ICBC, 2 allocated to IWBC)

^4^ Data were missing for 1 patient (allocated to ICBC)

^5^ Data were missing for 2 patients (allocated to ICBC)

^6^ Data were missing for 8 patients (5 allocated to ICBC, 3 allocated to IWBC)

^7^ Data were missing for 45 patients (24 allocated to ICBC, 21 allocated to IWBC)

^8^ Data were missing for 4 patients (2 allocated to ICBC, 2 allocated to IWBC)

^9^ Data were missing for 9 patients (5 allocated to ICBC, 4 allocated to IWBC)

^10^ Data were missing for 46 patients (24 allocated to ICBC, 22 allocated to IWBC)

^11^ Data were missing for 44 patients (23 allocated to ICBC, 21 allocated to IWBC)

Table S 12 Secondary outcomes and related data III: Chest and wound infections

| **Outcome (or related data)** | |  | **Randomised to ICBC (n=48)** | | **Randomised to IWBC (n=49)** | | **Overall**  **(n=97)** | |
| --- | --- | --- | --- | --- | --- | --- | --- | --- |
|  |  |  | **n** | **%** | **n** | **%** | **n** | **%** |
| POSTOPERATIVE INFECTIVE COMPLICATIONS | | | | |  |  |  |  |
| Infective chest or wound complication ^1^ | | (events/ patients) | 12/12 | 25.5% | 24/21 | 42.9% | 36/33 | 34.4% |
| **Confirmed infective chest or wound complications** ^1^ | | (events/ patients) | 7/7 | 14.9% | 14/12 | 24.5% | 21/19 | 19.8% |
|  | |  |  |  |  |  |  |  |
| Type of infective complication: | |  |  |  |  |  |  |  |
| Respiratory infection | |  | 11/47 | 23.4% | 21/49 | 42.9% | 32/96 | 33.3% |
|  | Infection confirmed |  | 7/11 | 63.6% | 12/21 | 57.1% | 19/32 | 59.4% |
| Superficial wound infection | |  | 1/47 | 2.1% | 2/49 | 4.1% | 3/96 | 3.1% |
|  | Infection confirmed |  | 0/1 | 0.0% | 1/2 | 50.0% | 1/3 | 33.3% |
| Mediastinitis | |  | 0/47 | 0.0% | 0/49 | 0.0% | 0/96 | 0.0% |
| Wound dehiscence requiring rewiring or treatment | |  | 0/47 | 0.0% | 1/49 | 2.0% | 1/96 | 1.0% |
|  | Infection confirmed |  |  |  | 1/1 | 100.0% | 1/1 | 100.0% |
| Other postoperative infection with antibiotic treatment | |  | 3/47 | 6.4% | 2/49 | 4.1% | 5/96 | 5.2% |
|  | Infection confirmed |  | 1/3 | 33.3% | 0/2 | 0.0% | 1/5 | 20.0% |
|  |  |  |  |  |  |  |  |  |
| Antibiotic treatment: details:- | |  |  |  |  |  |  |  |
| Number of courses of antibiotic treatment | | 0 | 32/47 | 68.1% | 25/49 | 51.0% | 57/96 | 59.4% |
|  |  | 1 | 7/47 | 14.9% | 13/49 | 26.5% | 20/96 | 20.8% |
|  | | 2 | 8/47 | 17.0% | 8/49 | 16.3% | 16/96 | 16.7% |
|  | | 3 | 0/47 | 0.0% | 1/49 | 2.0% | 1/96 | 1.0% |
|  | | 4 | 0/47 | 0.0% | 1/49 | 2.0% | 1/96 | 1.0% |
|  | | 6 | 0/47 | 0.0% | 1/49 | 2.0% | 1/96 | 1.0% |
| Duration of antibiotic treatment (days) ^2^ | | Median (IQR) | 5 | (3.0, 7.0) | 4 | (3.0, 7.0) | 5 | (3.0, 7.0) |
| Antibiotic given | |  |  |  |  |  |  |  |
|  | Amoxicillin |  | 1/15 | 6.7% | 1/24 | 4.2% | 2/39 | 5.1% |
|  | Cefuroxime |  | 0/15 | 0.0% | 1/24 | 4.2% | 1/39 | 2.6% |
|  | Co-amoxiclav |  | 3/15 | 20.0% | 7/24 | 29.2% | 10/39 | 25.6% |
|  | Flucloxacillin |  | 0/15 | 0.0% | 1/24 | 4.2% | 1/39 | 2.6% |
|  | Gentamicin |  | 0/15 | 0.0% | 1/24 | 4.2% | 1/39 | 2.6% |
|  | Linezolid |  | 0/15 | 0.0% | 1/24 | 4.2% | 1/39 | 2.6% |
|  | Meropenem |  | 0/15 | 0.0% | 1/24 | 4.2% | 1/39 | 2.6% |
|  | Metronidazole |  | 0/15 | 0.0% | 1/24 | 4.2% | 1/39 | 2.6% |
|  | Oseltamivir |  | 1/15 | 6.7% | 0/24 | 0.0% | 1/39 | 2.6% |
|  | Piperacillin |  | 1/15 | 6.7% | 0/24 | 0.0% | 1/39 | 2.6% |
|  | Piperacillin/Tazobactam |  | 14/15 | 93.3% | 21/24 | 87.5% | 35/39 | 89.7% |
|  | Teicoplanin |  | 2/15 | 13.3% | 5/24 | 20.8% | 7/39 | 17.9% |
|  | Vancomycin |  | 1/15 | 6.7% | 0/24 | 0.0% | 1/39 | 2.6% |
| INFECTIVE COMPLICATIONS DURING FOLLOW-UP (SERIOUS COMPLICATIONS ONLY) | | | | | | | | |
| Serious infective complications during follow-up (occurring post-discharge) | |  | 4/44 | 9.1% | 2/48 | 4.2% | 6/92 | 6.5% |
| Infective complications requiring antibiotics | |  | 2/44 | 4.5% | 1/48 | 2.1% | 3/92 | 3.3% |
| Infection site | |  |  |  |  |  |  |  |
| Wound | |  | 2/2 | 100.0% | 1/1 | 100.0% | 3/3 | 100.0% |
| Other infective complications: | |  |  |  |  |  |  |  |
|  | Respiratory infection |  | 2/44 | 4.5% | 1/48 | 2.1% | 3/92 | 3.3% |
|  | Superficial wound infection |  | 0/44 | 0.0% | 0/48 | 0.0% | 0/92 | 0.0% |
|  | Mediastinitis |  | 0/44 | 0.0% | 0/48 | 0.0% | 0/92 | 0.0% |
|  | Wound dehiscence requiring rewiring or treatment |  | 0/44 | 0.0% | 0/48 | 0.0% | 0/92 | 0.0% |
|  | Other postoperative infection with antibiotic treatment |  | 0/44 | 0.0% | 1/48 | 2.1% | 1/92 | 1.1% |

ICBC = intermittent cold blood cardioplegia, IWBC = intermittent cold blood cardioplegia, IQR = interquartile range

**Note:**

Secondary outcome indicated in bold.

^1^ Data were missing for 1 patient (allocated to ICBC)

^2^ Data were missing for 3 patients (allocated to IWBC)

Table S 13 Postoperative details

| Characteristic^1^ | |  | Randomised to ICBC (n=48) | | Randomised to IWBC (n=49) | | Overall  (n=97) | |
| --- | --- | --- | --- | --- | --- | --- | --- | --- |
|  | |  | n | % | n | % | n | % |
| POSTOPERATIVE DETAILS | |  |  |  |  |  |  |  |
| Core temperature on return from theatre (*°*C) | | mean (SD) | 36.4 | 0.8 | 36.5 | 1.0 | 36.5 | 0.9 |
| Fluid balance at 24h (mL) | | median (IQR) | 22 | (-112.0, 131.0) | 25 | (-153.0, 124.0) | 24 | (-134.5, 130.0) |
| POSTOPERATIVE BLOOD PRODUCTS | | |  |  |  |  |  |  |
| RBC | |  | 6/47 | 12.8% | 8/49 | 16.3% | 14/96 | 14.6% |
| Total postoperative RBC  (mL) | | median (IQR) | 225 | (70.0, 260.0) | 96 | (34.0, 120.0) | 110 | (50.0, 250.0) |
| FFP | |  | 1/47 | 2.1% | 2/49 | 4.1% | 3/96 | 3.1% |
| Total postoperative FFP  (mL) | | median (IQR) | 60 | (60.0, 60.0) | 150 | (150.0, 150.0) | 150 | (60.0, 150.0) |
| Platelets | |  | 1/47 | 2.1% | 3/49 | 6.1% | 4/96 | 4.2% |
| Total postoperative  platelets (mL) | | median (IQR) | 60 | (60.0, 60.0) | 140 | (53.0, 150.0) | 100 | (56.5, 145.0) |
| Cryoprecipitate | |  | 4/47 | 8.5% | 1/49 | 2.0% | 5/96 | 5.2% |
| Total postoperative  cryoprecipitate (mL) | | median (IQR) | 165 | (30.0, 350.0) | 75 | (75.0, 75.0) | 75 | (30.0, 300.0) |
| CHEST CLOSURE | |  |  |  |  |  |  |  |
| Chest closed on return from  theatre | |  | 46/47 | 97.9% | 46/49 | 93.9% | 92/96 | 95.8% |
| Location of chest closure if  chest not closed on return  from theatre | | PICU | 1/1 | 100.0% | 3/3 | 100.0% | 4/4 | 100.0% |
| REINTERVENTIONS | |  |  |  |  |  |  |  |
| Number of reinterventions | | 0 | 43/47 | 91.5% | 38/49 | 77.6% | 81/96 | 84.4% |
|  | | 1 | 3/47 | 6.4% | 9/49 | 18.4% | 12/96 | 12.5% |
|  | | 2 ^2^ | 1/47 | 2.1% | 2/49 | 4.1% | 3/96 | 3.1% |
|  | |  |  |  |  |  |  |  |
| Procedure type: | | Chest  reopened ^3^ | 0/47 | 0.0% | 1/49 | 2.0% | 1/96 | 1.0% |
|  | | Permanent  pacemaker | 2/47 | 4.3% | 8/49 | 16.3% | 10/96 | 10.4% |
|  | | Planned ^4^ | 1/47 | 2.1% | 0/49 | 0.0% | 1/96 | 1.0% |
|  | | Unplanned ^5^ | 2/47 | 4.3% | 4/49 | 8.2% | 6/96 | 6.3% |
|  | | Other ^6^ | 0/47 | 0.0% | 1/49 | 2.0% | 1/96 | 1.0% |
| Time from operation to re-intervention (days) | |  | 16 | (9.0, 17.0) | 10 | (5.0, 11.0) | 10 | (5.0, 16.0) |
| RBC | |  | 0/5 | 0.0% | 4/13 | 30.8% | 4/18 | 22.2% |
| Total RBC (mL) | | median (IQR) |  |  | 55 | (45.0, 110.0) | 55 | (45.0, 110.0) |
| FFP | |  | 0/5 | 0.0% | 1/13 | 7.7% | 1/18 | 5.6% |
| Total FFP (mL) | | median (IQR) |  |  | 150 | (150.0, 150.0) | 150 | (150.0, 150.0) |
| Platelets | |  | 0/5 | 0.0% | 4/13 | 30.8% | 4/18 | 22.2% |
| Total platelets (mL) | | median (IQR) |  |  | 115 | (47.5, 175.0) | 115 | (47.5, 175.0) |
| Cryoprecipitate | |  | 0/5 | 0.0% | 3/13 | 23.1% | 3/18 | 16.7% |
| Total cryoprecipitate  (mL) | | median (IQR) |  |  | 50 | (50.0, 60.0) | 50 | (50.0, 60.0) |
| POSTOPERATIVE INOTROPES | | |  |  |  |  |  |  |
| Dopamine | |  | 37/47 | 78.7% | 40/49 | 81.6% | 77/96 | 80.2% |
|  | Dose (µg/kg/min) | Median (IQR) | 5.0 | (5.00, 5.00) | 5.0 | (5.00, 5.00) | 5.0 | (5.00, 5.00) |
| Milrinone | |  | 43/47 | 91.5% | 45/49 | 91.8% | 88/96 | 91.7% |
|  | Dose (µg/kg/min) | Median (IQR) | 0.5 | (0.50, 0.50) | 0.5 | (0.50, 0.50) | 0.5 | (0.50, 0.50) |
| Adrenaline | |  | 4/47 | 8.5% | 10/49 | 20.4% | 14/96 | 14.6% |
|  | Dose (µg/kg/min) | Median (IQR) | 0.1 | (0.03, 0.10) | 0.1 | (0.10, 0.10) | 0.1 | (0.10, 0.10) |
| Noradrenaline | |  | 2/47 | 4.3% | 7/49 | 14.3% | 9/96 | 9.4% |
|  | Dose (µg/kg/min) | Median (IQR) | 0.1 | (0.02, 0.10) | 0.1 | (0.10, 0.10) | 0.1 | (0.10, 0.10) |
| Dobutamine | |  | 0/47 | 0.0% | 0/49 | 0.0% | 0/96 | 0.0% |
| Vasopressin | |  | 2/47 | 4.3% | 1/49 | 2.0% | 3/96 | 3.1% |
|  | Dose (µg/kg/min) | Median (IQR) | 0.0032 | (0.0015, 0.0050) | 0.0005 | (0.0005, 0.0005) | 0.0015 | (0.0005, 0.0050) |
| Sodium nitroprusside | |  | 4/47 | 8.5% | 1/49 | 2.0% | 5/96 | 5.2% |
|  | Dose (µg/kg/min) | Median (IQR) | 0.4 | (0.28, 0.74) | 2.0 | (2.00, 2.00) | 0.5 | (0.30, 1.00) |
| Milrinone (single strength) | |  | 1/47 | 2.1% | 0/49 | 0.0% | 1/96 | 1.0% |
|  | Dose (µg/kg/min) | Median (IQR) | 0.5 | (0.50, 0.50) |  |  | 0.5 | (0.50, 0.50) |
| Any inotrope restarted | |  | 3/47 | 6.4% | 4/49 | 8.2% | 7/96 | 7.3% |

ICBC = intermittent cold blood cardioplegia, IWBC = intermittent cold blood cardioplegia, SD = standard deviation, IQR = interquartile range, RBC = red blood cells, FFP = fresh frozen plasma, PICU = paediatric intensive care unit.

**Note:**

^1^ Unless otherwise stated, data were missing for 1 patient (allocated to ICBC)

^2^ One patient (allocated to IWBC) had two procedures (permanent pacemaker insertion and *relief of LVOT obstruction) performed as part of a single reintervention.*

^3^ Chest was reopened for bleeding in one patient (allocated to IWBC)

^4^ *Planned procedure was pacemaker change.*

^5^ U*nplanned/semi-planned procedures were: -*

*ICBC: atrioventricular valve replacement, pericardial drain insertion;*

*IWBC: Relief of LVOT obstruction, pericardial drain insertion, repair of left/right atrioventricular valve, and repeat*

*right ventricle to pulmonary artery conduit*

^6^ *other procedure was: diagnostic cardiac catheterisation. One further procedure of ‘chest exploration‘ was also recorded but this was not considered to be major enough to be classed as a reintervention as the chest was still open from the original surgery.*

Table S 14 Hospital re-admissions within 3 months of operation

|  | Randomised to ICBC (n=48) | | Randomised to  IWBC (n=49) | | Overall  (n=97) | |
| --- | --- | --- | --- | --- | --- | --- |
| Event type | n | % | n | % | n | % |
| Total hospital readmissions  (events/patients) ^1^ | 16/9 | 20.5% | 13/7 | 14.6% | 29/16 | 17.4% |
| ARRHYTHMIAS |  |  |  |  |  |  |
| Atrial Tachycardia ^2^ | 0/44 | 0.0% | 1/48 | 2.1% | 1/92 | 1.1% |
| OTHER CARDIAC DISORDERS |  |  |  |  |  |  |
| Cardiogenic shock ^2^ | 0/44 | 0.0% | 1/48 | 2.1% | 1/92 | 1.1% |
| HAEMODYNAMIC SUPPORT |  |  |  |  |  |  |
| ECMO | 0/44 | 0.0% | 1/48 | 2.1% | 1/92 | 1.1% |
| Low cardiac output | 0/44 | 0.0% | 1/48 | 2.1% | 1/92 | 1.1% |
| PERICARDIAL EFFUSION |  |  |  |  |  |  |
| Pericardial effusion ^3^ | 0/44 | 0.0% | 2/48 | 4.2% | 2/92 | 2.2% |
| RESPIRATORY COMPLICATIONS |  |  |  |  |  |  |
| Respiratory presentation ^2 4^ | 3/44 | 6.8% | 0/48 | 0.0% | 3/92 | 3.3% |
| NEUROLOGICAL COMPLICATIONS |  |  |  |  |  |  |
| Somnolence ^2^ | 1/44 | 2.3% | 0/48 | 0.0% | 1/92 | 1.1% |
| Seizure-like phenomenon ^2^ | 1/44 | 2.3% | 0/48 | 0.0% | 1/92 | 1.1% |
| GASTROINTESTINAL COMPLICATIONS |  |  |  |  |  |  |
| Vomiting ^2^ | 1/44 | 2.3% | 0/48 | 0.0% | 1/92 | 1.1% |
| INFECTIVE COMPLICATIONS |  |  |  |  |  |  |
| Urinary tract infections ^2 5^ | 0/44 | 0.0% | 1/48 | 2.1% | 1/92 | 1.1% |
| Viral rash ^2^ | 1/44 | 2.3% | 0/48 | 0.0% | 1/92 | 1.1% |
| Respiratory infection ^6^ | 4/44 | 9.1% | 1/48 | 2.1% | 5/92 | 5.4% |
| Infective wound complications (antibiotic courses) | 2/44 | 4.5% | 1/48 | 2.1% | 3/92 | 3.3% |
| Other post operative infection with antibiotic treatment | 0/44 | 0.0% | 1/48 | 2.1% | 1/92 | 1.1% |
| OTHER COMPLICATIONS |  |  |  |  |  |  |
| Pyrexia ^2^ | 1/44 | 2.3% | 0/48 | 0.0% | 1/92 | 1.1% |
| Residual anatomical abnormalities (requiring surgery) | 0/44 | 0.0% | 1/48 | 2.1% | 1/92 | 1.1% |

ICBC = intermittent cold blood cardioplegia, IWBC = intermittent cold blood cardioplegia, ECMO, extracorporeal membrane oxygenation

Note*:*

^1^ Data were missing for 5 participants (4 allocated to ICBC, 1 allocated to IWBC)

^2^ These events were not listed in the study protocol as expected events – further details are given in Table S 15

*^3^ 1 participant allocated to IWBC had three separate admissions with pericardial effusion*

*^4^ 1 participant had respiratory distress, 1 participant had wheezing and 1 participant had three separate admissions presenting with dyspnoea, stridor and wheezing respectively*

^5^ Participant received alternative treatment to that allocated

^6^ Two of these events (bronchiolitis, chest infection) were not listed in the study protocol as expected events – further details are given in Table S 15

Table S 15 Details of hospital readmissions (within 3 months of operation) for reasons not listed in the protocol as expected events

| **ID** | **Allocation Group** | **MedDRA preferred term** | **Reason classified as serious** | **Maximum Intensity** | **Relatedness to treatment** | **Outcome** |
| --- | --- | --- | --- | --- | --- | --- |
| 1 | ICBC | Pyrexia | Required hospitalisation | Moderate | Not related | Resolved, no sequelae |
| 1 | ICBC | Seizure like phenomena | Required hospitalisation | Moderate | Not related | Resolved, no sequelae |
| 2 | ICBC | Croup infectious | Required hospitalisation | Mild | Not related | Resolved, no sequelae |
| 3 | ICBC | Somnolence | Required hospitalisation | Mild | Not related | Resolved, no sequelae |
| 4 | ICBC | Dyspnoea | Required hospitalisation | Mild | Not related | Resolved, no sequelae |
| 4 | ICBC | Wheezing | Required hospitalisation | Mild | Not related | Resolved, no sequelae |
| 4 | ICBC | Vomiting | Required hospitalisation | Moderate | Not related | Resolved, no sequelae |
| 4 | ICBC | Stridor | Required hospitalisation | Mild | Not related | Resolved, no sequelae |
| 5 | ICBC | Wheezing | Required hospitalisation | Moderate | Not related | Resolved, no sequelae |
| 6 | ICBC | Bronchiolitis | Required hospitalisation | Moderate | Not related | Resolved, no sequelae |
| 7 | ICBC | Respiratory distress | Required hospitalisation | Mild | Not related | Resolved, no sequelae |
| 7 | ICBC | Viral rash | Required hospitalisation | Mild | Not related | Resolved, no sequelae |
| 8 | IWBC | Urinary tract infections | Required hospitalisation | Mild | Not related | Resolved, no sequelae |
| 9 | IWBC | Atrial tachycardia | Required hospitalisation | Mild | Not related | Resolved, no sequelae |
| 10 | IWBC | Cardiogenic shock | Required hospitalisation and ECMO, Life-threatening | Severe | Unlikely to be related | Resolved, no sequelae |

ICBC = intermittent cold blood cardioplegia, IWBC = intermittent cold blood cardioplegia, ECMO = extracorporeal membrane oxygenation.
